# Topological potentials guiding protein self-assembly

**DOI:** 10.1371/journal.pcbi.1014709

**Published:** 2026-09-03

**Authors:** Ivan L. A. Spirandelli, Arnur Nigmetov, Dmitriy Morozov, Myfanwy E. Evans

**Affiliations:** 1 Institute for Mathematics, University of Potsdam, Potsdam, Brandenburg, Germany; 2 Scientific Data Division, Lawrence Berkeley National Laboratory, Berkeley, California, United States of America; 3 Applied Mathematics and Computational Research Division, Lawrence Berkeley National Laboratory, Berkeley, California, United States of America; University of Massachusetts Amherst, UNITED STATES OF AMERICA

## Abstract

The simulated assembly of molecular building blocks into functional complexes is central to computational biology and materials science. Protein-assembly simulations, driven by short-range nonpolar interactions, can in principle reach their biologically correct structures, but rugged energy landscapes often trap simulations in non-functional local minima. We introduce a long-range topological potential, quantified by weighted total persistence, and combine it with the morphometric approach to solvation free energy. Across four protein systems, this combination increases assembly success rates by up to sixteen-fold and enables assembly in cases that otherwise fail. Unlike previous topology-based approaches, our method uses topological measures as an active energetic bias rather than a descriptive tool. Depending only on atom geometry, the method extends in principle to other self-assembling systems, offering a general strategy for overcoming kinetic barriers in molecular simulations.

## Author summary

We developed a new computational method that helps simulate how proteins assemble into functional complexes. Proteins often need to come together in precise arrangements to function, but computer simulations of this process frequently get stuck in incorrect configurations due to the complexity of the energy landscape. Our key insight is that tools from computational topology, specifically persistent homology, can be used not just to analyze molecular shapes after the fact, but as an active guiding force within the simulation itself. We define a topological potential that captures shape information over long distances, complementing a geometric model of solvation free energy that drives assembly at close contact. When we combine both potentials, the topological term acts as a funnel that pulls protein subunits into roughly the right arrangement, while the geometric term handles precise docking. We tested this on four different protein systems, including components of the tobacco mosaic virus, hepatitis B, SARS-CoV-2, and the human immune system, and found that our combined approach dramatically improves assembly success rates, increasing them by up to sixteen-fold compared to using the geometric model alone.

## Introduction

In silico assembly of molecular building blocks into functional complexes is a central challenge in biology and materials science. A primary obstacle is the problem of kinetic trapping [1,2]: Even when the functional native structure represents the minimum of the energy function that governs the simulation, the vastness of the configurational landscape, containing many nonfunctional local minima, can prevent simulations from discovering it on a feasible timescale. A successful simulation methodology must therefore not only be based on an accurate energy function that identifies the native state, but also navigate its landscape efficiently to avoid dead-end assembly pathways.

Here, we introduce a long-range potential based on the principles of computational topology to act as a guiding bias. Topological data analysis, particularly persistent homology [3,4], is utilized to solve optimization tasks in machine learning contexts [5,6], and has been widely used to analyze static and dynamic molecular data [7–10] and nanomaterials [11,12] post hoc. We use it here as an active component within the simulation of protein assembly.

The physically motivated core of our simulations is the *morphometric approach to solvation free energy* [13,14]. It is an implicit solvent model [15,16] and a sophisticated implementation, describing non-polar contributions to the solvation free energy as a linear combination of four geometric measures: the excluded volume *V*, solvent-accessible surface area *A*, integrated mean curvature *C*, and integrated Gaussian curvature *X* of the solvent-accessible surface [17–19]. This method has proven highly accurate for calculating solvation properties of complex biomolecules [20–23] and assembles exotic geometries for generic solutes [24,25]. For simulation of macromolecules, we augment it with a soft-sphere overlap penalty *L*, yielding a total short-range geometric potential:


$$\begin{array}{c} F_{sol}^{*}=pV+\sigma A+\kappa C+\overset{―}{\kappa }X+\phi L. \end{array}$$ (1)

The prefactors for the geometric terms are derived from fundamental measure theory [26], where *p* is pressure, σ is surface tension, and κ and $\overset{―}{\kappa }$ are bending rigidities [14]. The weight ϕ is empirically determined and, in combination with *L*, acts both as a simple model of repulsion and as a relaxation of the shape of the modeled molecules.

The excluded volume *V* describes the volume inaccessible to solvent molecule centers. The surface area *A* captures the area available to solvent molecule centers as close to the solute as possible. The integrated mean curvature *C* measures the total bending of the solvent-accessible surface. The integrated Gaussian curvature *X* is a topological measure equal to 4π times the Euler characteristic of the union of balls, hence measuring connectivity, loops and voids of the configuration.

We calculate the prefactors utilizing the White Bear functional mark II [26] from density functional theory, giving us values for a hard-sphere solvent depending on solvent radius $r_{s}$ and packing fraction η, which we set to 1.4Å and 0.3665 respectively to mimic the properties of water. As such, the effective attraction that drives the assembly of the solutes is entropic in origin.

Previous work has shown that for tobacco mosaic virus (TMV) subunits, the assembly observed experimentally corresponds to the minimum of $F_{sol}^{*}$ obtained via Random Walk Metropolis simulations [27]. Random Walk Metropolis explores the configuration space by perturbing the current configuration to propose a new one. A proposal that lowers the energy is always accepted, whereas a proposal that raises the energy is accepted only with a probability that decreases as the energy increase grows, and is otherwise rejected. This makes it a powerful algorithm for exploring the configuration space and locating thermodynamically stable states. It does not, however, model dynamics, and no kinetic accessibility between successive states can be inferred from the sequence of configurations it produces.

The morphometric approach is inherently short-ranged, as its geometric measures are defined by a local solvent probe (e.g., $r_{s}=1.4Å$ for water). Consequently, its energy landscape is largely flat until the subunits are in close contact, at which point it becomes rugged. A simulation driven only by $F_{sol}^{*}$ performs a largely random search until protein subunits interact at a close range. Here, the simulations find local minimizers that are sometimes difficult to escape from, preventing further exploration and correct assembly. This highlights a kinetic, rather than thermodynamic, hurdle that the model has to overcome in a simulation context.

In physical systems long-range interactions such as electrostatics [16] and long-range hydration forces [28] pull the proteins together and correctly align them. The sum of these effects can be interpreted as a long-range shape matching effect. The biasing potential we introduce in the following does not have a direct physical interpretation but acts as a long-range shape matching potential and in this sense is a similar mechanism to long-range physical interactions.

**Fig 1:**
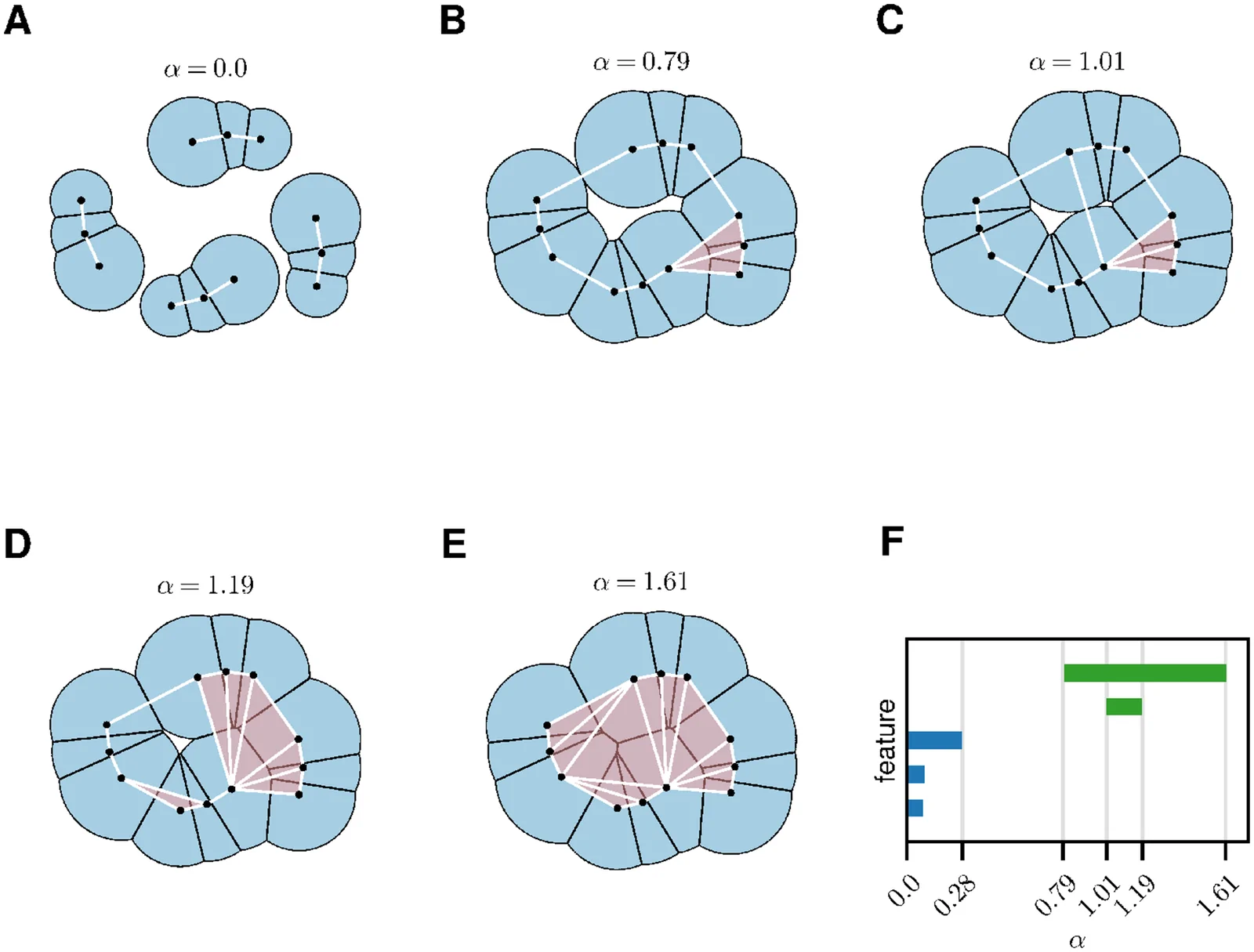
Alpha filtration and persistence. Weighted Alpha complexes for a system of four toy shapes in two dimensions at increasing values of α (A-E). Vertices are shown in black, edges in white and triangles in red; the dual intersection of the balls with the weighted Voronoi diagram is shown in blue. At α=0 the shapes carry their solvent-accessible radii (A); as α grows, the space between them is progressively filled (B-E). Panel F shows the resulting persistence barcode: each bar is a topological feature and runs from its birth *b* to its death *d* along the α-axis. Blue bars are zero-dimensional features (connected components) and green bars one-dimensional features (loops). The length of a bar, d−b, is its persistence, and the total persistence $P_{i}$ is the sum of the lengths of all *i*-dimensional bars. We only show the features of finite persistence.

The calculation of morphometric measures already relies on weighted Alpha complexes [29–32], which naturally provide a multi-scale representation of shape known as a filtration (see Fig 1). This Alpha-filtration is constructed on the union of atom centers of the simulated molecules. Persistent homology is the tool for quantifying topological features within such a filtration.

We define a topological potential, 𝒯, as a weighted sum of total persistence measures:


$$\begin{array}{c} 𝒯=\lambda_{0}P_{0}+\lambda_{1}P_{1}+\lambda_{2}P_{2}, \end{array}$$ (2)

where $P_{i}=\sum_{(b,d)\in Dgm_{i}}(d-b)$ is the total persistence of all *i*-dimensional features (birth-death pairs) in the system’s persistence diagram $Dgm_{i}$ and the $\lambda_{i}$ are scalar weights.

Consider the persistence barcode in Fig 1F. As the scale α increases, the union of balls grows: initially separate components merge, and loops between the shapes form and later fill in. Each such feature is recorded as a bar from its birth *b*, the scale at which it appears, to its death *d*, the scale at which it disappears, and its persistence d−b measures the range of scales over which it survives. Concretely, the long green bar corresponds to the central loop that forms in panel B and persists until it is filled in panel E, while the short green bar corresponds to the short-lived loop that appears to the right of it in panel C and closes again by panel D. The total persistence $P_{i}$ in Eq 2 is exactly the summed length of all finite persistence *i*-dimensional bars, so 𝒯 is a weighted readout of this barcode. The zero-dimensional persistence *P*_0_ is small when the distinct molecules are close together. *P*_1_ and *P*_2_ track the total persistence of loops and voids enclosed between the molecules. Note that no voids exist in the two-dimensional example in Fig 1. Total persistence of loops and voids can be large when few features persist for a long time or many features persist for a short time. The weights $\lambda_{i}$ tune how strongly these terms are maximized or minimized during assembly.

In the results section we determine promising candidates for the $\lambda_{i}$ based on a grid search in which we explore for which parameters the topological potential alone obtains minima that are energetically close to true assemblies of TMV protein subunits. The initial boundaries of the grid are chosen based on observations about the total persistence measures as well as theoretical considerations. The grid is then extended to ensure that the most promising region of the scan lies away from the boundaries.

The potential captures some shape information over arbitrary ranges, suggesting a synergistic strategy in which the two potentials play complementary roles. The long-range topological term, 𝒯, can create a guiding funnel within the energy landscape, pulling subunits into the correct general vicinity and orientation. The short-range geometric potential, $F_{sol}^{*}$, then acts as a refiner, selecting the precise low-energy binding interface. In this work, we test this principle by simulating dimer and trimer assembly of various systems governed by a combined potential:


$$\begin{array}{c} E_{comb}=\mu F_{sol}^{*}+(1-\mu )𝒯. \end{array}$$ (3)

We show that this approach, which combines local physical accuracy with global topological guidance, enhances the purely geometric model, increasing the simulation success rate for the TMV and SARS-CoV-2 ORF9b proteins by more than an order of magnitude and further making the successful assembly of a hepatitis B core protein and an extracellular fragment of the human CD40 ligand possible on the simulated timescales. This result validates the use of topology not just as a post-hoc analytical tool, but as an active biasing potential to solve kinetic challenges in molecular simulation.

## Results

We begin by discussing how $F_{sol}^{*}$ and 𝒯 are correlated and analyze how persistence measures develop over the course of simulated assemblies driven only by $F_{sol}^{*}$. We do this to motivate the use of topological measures in our simulations using the inherent relationships between geometric and topological measures. We then investigate the minimizers of 𝒯 for different combinations of $\left(\lambda_{0},\lambda_{1},\lambda_{2}\right)$. Finally, we compare different mixtures of $F_{sol}^{*}$ and 𝒯 and how the inclusion of the topological potential improves the simulation results.

### Geometry-topology correlations

We compare the topological and geometric potential, calculating 𝒯 for a sequence of configurations of two TMV capsid protein subunits, obtained via a *successful* simulation run driven by the geometric potential alone. Here, successful means that the two subunits are assembled in a configuration similar to one observed experimentally, as the simulation reaches the energy minimum. Let $S_{ca}$ be an experimentally determined configuration of two correctly assembled protein subunits. Let *S* be a configuration obtained by simulation. We say that *S* and $S_{ca}$ are similar if $d_{\text{RMSD}}(S,S_{ca})<2.5Å$. See Materials and Methods for an explanation of the distance measure *d*_RMSD_. A visualization of the correct assembly of two protein subunits is shown in Fig 3A. The simulation is driven by a Random Walk Metropolis algorithm; see Materials and Methods.

### Calculating total persistence for a successful simulation run

**Fig 2:**
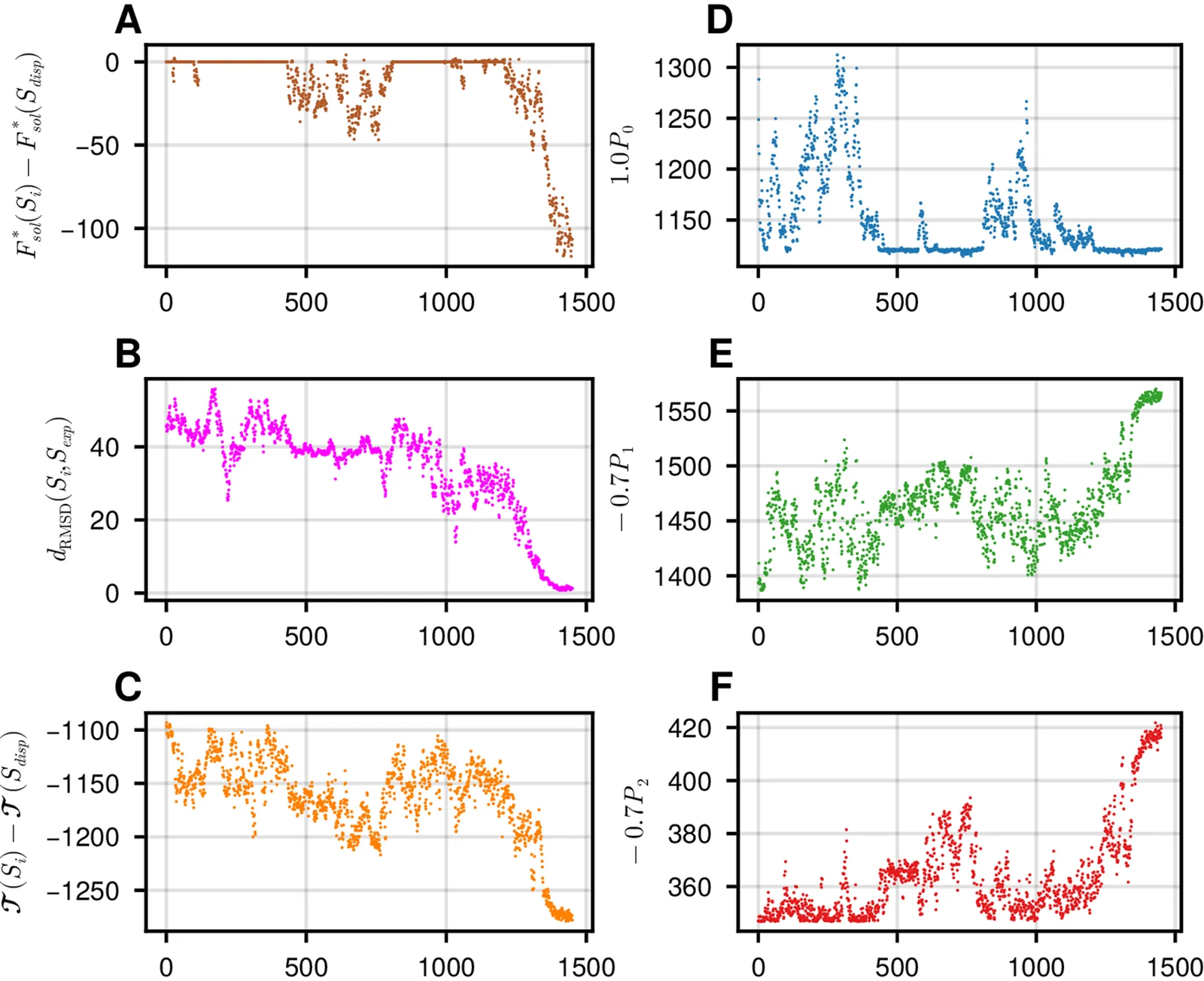
Comparing potentials. Comparing potentials of a sequence of states obtained via a simulation driven by the geometric potential $F_{sol}^{*}$. The geometric potential itself (A). A goodness of fit measure with respect to the correct assembly of two TMV proteins (B). The weighted sum $𝒯=0.5P_{0}-0.35P_{1}-0.35P_{2}$ (C). The total persistence in dimension zero (D), dimension one (E) and dimension two (F).

Fig 2A shows how $F_{sol}^{*}$, Eq 1, changes over the course of approximately 1500 steps of a simulated assembly pathway. Here, one step corresponds to an accepted proposal of the Random Walk Metropolis algorithm, i.e., a change in the configuration of the simulated molecules. The signal is flat for large stretches of the simulation because the geometric potential is short-ranged and only exhibits changes in value when the solvent-accessible surfaces of the simulated proteins overlap. There are several small valleys in the energy landscape, which correspond to configurations where the subunits are close. They detach again several times before finding a sequence of moves leading to a steep decline in energy and a local minimum, corresponding to configurations that are close to experimentally observed assembly. Fig 2B shows the goodness-of-fit measure *d*_RMSD_ that tracks how close to correct assembly the current configuration is. We can see that *d*_RMSD_ is more stable in the regions where the subunits overlap and oscillates in the regions where $F_{sol}^{*}$ is flat. Around step *i* = 1000 there is a drop in *d*_RMSD_ corresponding to the protein subunits being correctly aligned but not yet in contact. As $F_{sol}^{*}$ reaches its minimum, so does *d*_RMSD_, which fluctuates around a low value indicating proximity to the correct assembly.

Fig 2D-2F shows the weighted total persistence measures for dimensions zero, one and two. The chosen weights are $\lambda_{0}=1.0,\lambda_{1}=-0.7,\lambda_{2}=-0.7$. These weights correspond to the best performance increase achieved by mixing the geometric and topological potentials. The sum of all three, i.e., ${𝒯}_{\left(1.0,-0.7,-0.7\right)}$, is shown in Fig 2C. The total persistence of the zeroth homology group is plotted in blue and is minimal as soon as the proteins are close together because then the zero-dimensional hole between the two rigid bodies is closed. It is interesting to note that *P*_0_ is flat where $F_{sol}^{*}$ is not and vice versa. In addition, it seems that *P*_2_ and $F_{sol}^{*}$ are correlated and that *d*_RMSD_ and ${𝒯}_{\left(1.0,-0.7,-0.7\right)}$ share similar behavior.

To quantify these observations, we calculate the Pearson coefficient [33] *r* of the different functions. We list the Pearson coefficients of different measures of sequences of configurations of two TMV capsid proteins, in Table 1. In the table, 𝒯 refers to ${𝒯}_{\left(1.0,-0.7,-0.7\right)}$. The Pearson coefficient measures the strength and direction of the linear relationship between two quantities, ranging from −1 (perfectly anti-correlated) through 0 (no linear relationship) to +1 (perfectly correlated). Here it is informative since it indicates that the topological potential is correlated to *d*_RMSD_, pointing toward an exploitable signal to drive simulations toward the assembled state. It furthermore indicates which signs for the weights for total persistence per dimension are likely appropriate.

**Table 1:** Linear correlations between different measures.

	$F_{sol}^{*}$	*d*_RMSD_	𝒯	*P*_0_	*P*_1_	*P*_2_
$F_{sol}^{*}$	1.0	0.71	0.86	0.38	−0.8	−0.91
*d*_RMSD_	0.71	1.0	0.76	0.44	−0.7	−0.75
𝒯	0.86	0.76	1.0	0.42	−0.98	−0.95
*P*_0_	0.38	0.44	0.42	1.0	−0.26	−0.46
*P*_1_	−0.8	−0.7	−0.98	−0.26	1.0	0.87
*P*_2_	−0.91	−0.75	−0.95	−0.46	0.87	1.0

Linear correlations that stand out in particular are the high correlations of ${𝒯}_{\left(1.0,-0.7,-0.7\right)}$ to both *d*_RMSD_ and $F_{sol}^{*}$, which are higher than the correlation between *d*_RMSD_ and $F_{sol}^{*}$ themselves. The correlation of *d*_RMSD_ to ${𝒯}_{\left(1.0,-0.7,-0.7\right)}$ is higher (in absolute terms) than to each individual persistence measure. Furthermore, there is a high negative correlation between $F_{sol}^{*}$ and *P*_2_ of −0.91. The measures highly correlated in Table 1 match the plots in Fig 2 that exhibit similar trends.

A final key observation is that ${𝒯}_{\left(1.0,-0.7,-0.7\right)}$ and the corresponding weighted persistence measures are at or close to their respective minima as the simulation reaches its assembled state. This in combination with the relatively high linear correlation between *d*_RMSD_ and ${𝒯}_{\left(1.0,-0.7,-0.7\right)}$ motivates further analysis of the topological potential.

### Optimizing persistence

We investigate the self-assembly of two and three subunits of the TMV protein under minimization of topological potential 𝒯. Inferring promising weights for 𝒯 from the correlation of $F_{sol}$ and *d*_RMSD_ with the total persistence measures given in Table 1, we analyze the parameter space given by $\lambda_{0}=1.0$, $\lambda_{1}\in [-2.0,1.0]$ and $\lambda_{2}\in [-2.0,1.0]$. We run both Random Walk Metropolis and simulated annealing simulations in a grid with step size 0.1 in the parameter space. Let 𝒮 be the set of all minimal configurations obtained during one of the simulations. For each point in the parameter space, we then check which configuration in 𝒮 has the lowest topological potential. We then compare the topological potential of each minimizer to that of a correctly assembled state. We calculate


$$\begin{array}{c} d_{z}=\min_{S\in {𝒮}_{exp}}\log({𝒯}_{z}(S)-{𝒯}_{z}(S_{z})). \end{array}$$

**Fig 3:**
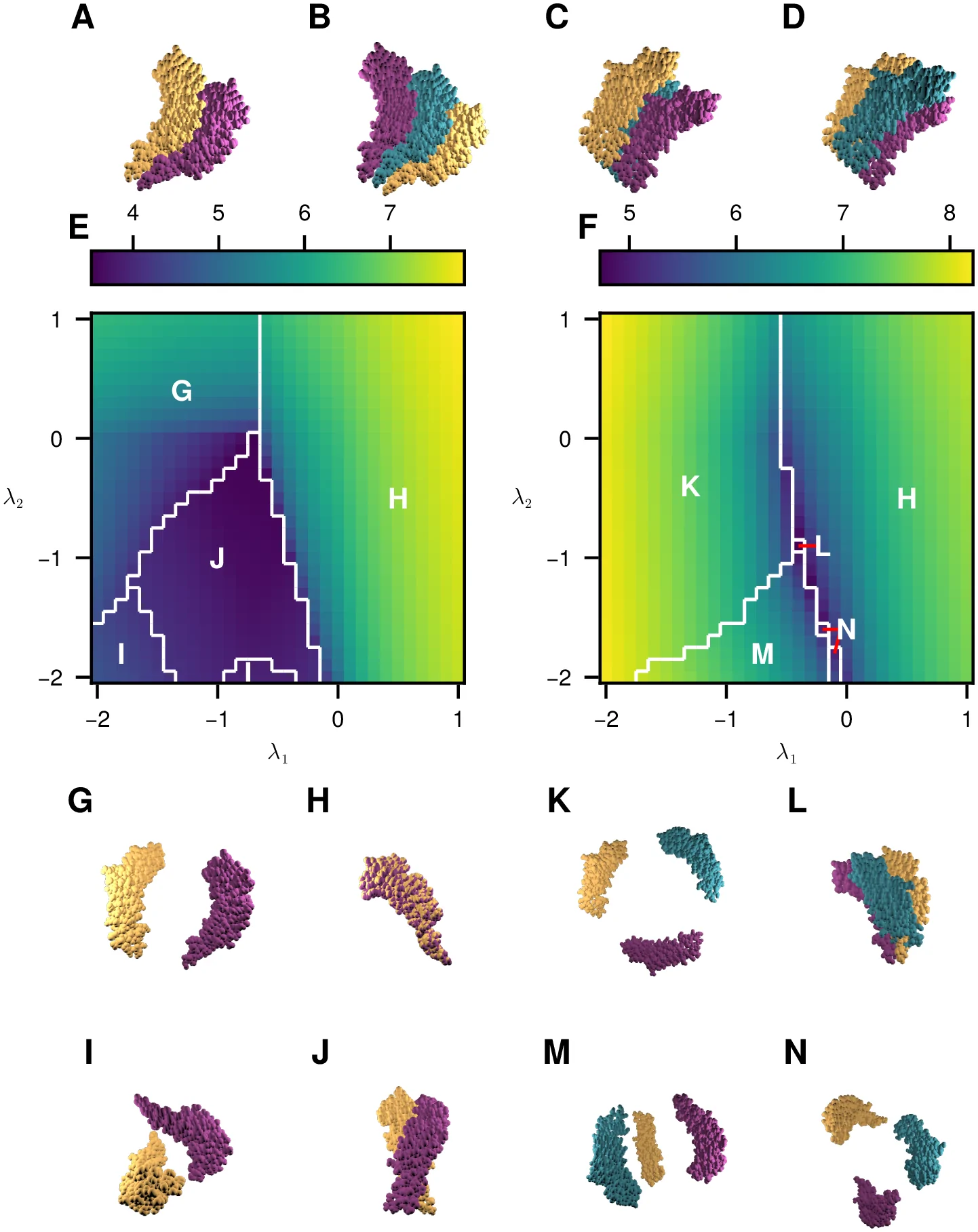
Topological potential of correct assemblies versus 𝒯 minimizers. Agreement between correctly assembled reference states and the minimizers of the topological potential 𝒯 across the weight parameter space. Render of the two correctly assembled TMV subunits used as the ground truth in the two-subunit case (A). Renders of the three configurations of three TMV subunits that we consider correctly assembled (B-D). Heatmaps over the weight plane $\left(\lambda_{1},\lambda_{2}\right)$, with $\lambda_{0}=1.0$ fixed (E,F). The color encodes $d_{z}=\log({𝒯}_{z}(S)-{𝒯}_{z}(S_{z}))$, the logarithm of the difference in topological potential between a correct assembly *S* and the minimizer $S_{z}$ found at weights $z=(\lambda_{0},\lambda_{1},\lambda_{2})$. Darker colors indicate a smaller difference, i.e., minimizers whose topological potential lies closer to that of the correct assembly. Groups of topological potential minimizers corresponding to the marked regions of the heatmaps (G-N); the minimizers closest to the correct assembly are J and L.

Here, $z=(\lambda_{0},\lambda_{1},\lambda_{2})$ refers to a point in the parameter space and $S_{z}$ to the topological potential minimizer at *z*. ${𝒮}_{exp}$ is the set of assemblies observed experimentally. In the case of two subunits, ${𝒮}_{exp}$ contains the single configuration shown in Fig 3A. In the case of three subunits, ${𝒮}_{exp}$ contains the configurations shown in Fig 3B-3D.

The resulting differences in topological potential $d_{z}$ for two and three subunits in the parameter space are plotted in Fig 3E and 3F. Dark colors correspond to the region in which the differences between ground truth and found minimizer are small. The bounded regions in the plots correspond to different groups of topological potential minimizers that we grouped by distance measurement *d*_RMSD_ and then manually aggregated further.

### Phases for two subunits

In the case of two subunits, we can distinguish four large regions. One in which the subunits overlap almost perfectly is a large region to the right of the phase diagram, corresponding to configurations as shown in subfigure H. The second group consists of configurations in which the protein subunits do not touch. They appear in the upper left and lower left corners of the phase diagram and in the center bottom. We note that on the top left the subunits appear to form a circle between them, while on the bottom they appear to form a ball. This is not surprising considering that the one-dimensional persistence features contribute relatively more to the top-left, while the two-dimensional features, to the bottom.

The final region lies roughly in the center of the phase diagram and contains configurations that touch and overlap slightly. None of the minimizing configurations is correctly assembled, although individual simulations did converge to a state close to correct assembly, which implies that it is a local minimizer.

### Phases for three subunits

The same images for three subunits are shown in Fig 3K-3N. Here, the region of non-touching subunits is much larger and roughly falls into three categories: configurations in which a circle forms between the subunits as in subfigure K; a ball, as in subfigure M; and finally a spiral configuration, as in subfigure N.

On the right side of the phase diagram there is the region with overlapping subunits, which is a bit smaller compared to the phase diagram of two subunits. This is caused by the possibility of more persistence features in dimensions one and two, counteracting the pull of minimizing zero-dimensional persistence. We have again marked this region with H, but note that here, three subunits overlap instead of two.

We again have a region in which the subunits touch but do not overlap completely, although it has almost disappeared. Interestingly, for this configuration two of the subunits are assembled in a way that corresponds to the correct assembly of two subunits, while the third one lies on top, with some overlap.

Considering the difference $d_{z}$ between the correct assembly and the topological potential minimizers, we see that for two subunits the region J, where the subunits touch but do not overlap corresponds to a dark area, that is, a small difference between the ground truth and the topological potential minimizer. For three subunits, this region of small $d_{z}$ values lies exactly at the boundary between the regions of minimizing configurations that do and do not overlap. The single point in the parameter space at $\lambda_{1}=-0.4$ and $\lambda_{2}=-0.9$ where the minimizer of three subunits touches but does not overlap is also the point in the parameter space with the lowest distance between ground truth and the topological potential minimizer.

### Combining geometry and topology

We will now compare Random Walk Metropolis simulations in which a combination of geometric and topological potential is minimized. We recall the combined potential, Eq 3:


$$\begin{array}{c} E_{comb}=\mu F_{sol}^{*}+(1-\mu )𝒯, \end{array}$$

where μ∈[0,1] is the interpolation between geometric and topological potential.

The measure in which we are interested is the success rate. Recall that a configuration *S* is correctly assembled if $d_{\text{RMSD}}(S,S_{ca})<2.5Å$, for $S_{ca}$ denoting the experimentally observed assembly. Let 𝒮 be a set of minimal configurations of a set of simulations, and let


$$\begin{array}{c} {𝒮}^{*}=\{S\in 𝒮|d_{\text{RMSD}}(S,S_{ca})<2.5Å\} \end{array}$$

be the set of successful simulations. We define the ratio $\frac{|{𝒮}^{*}|}{|𝒮|}$ as the success rate of 𝒮.

**Fig 4:**
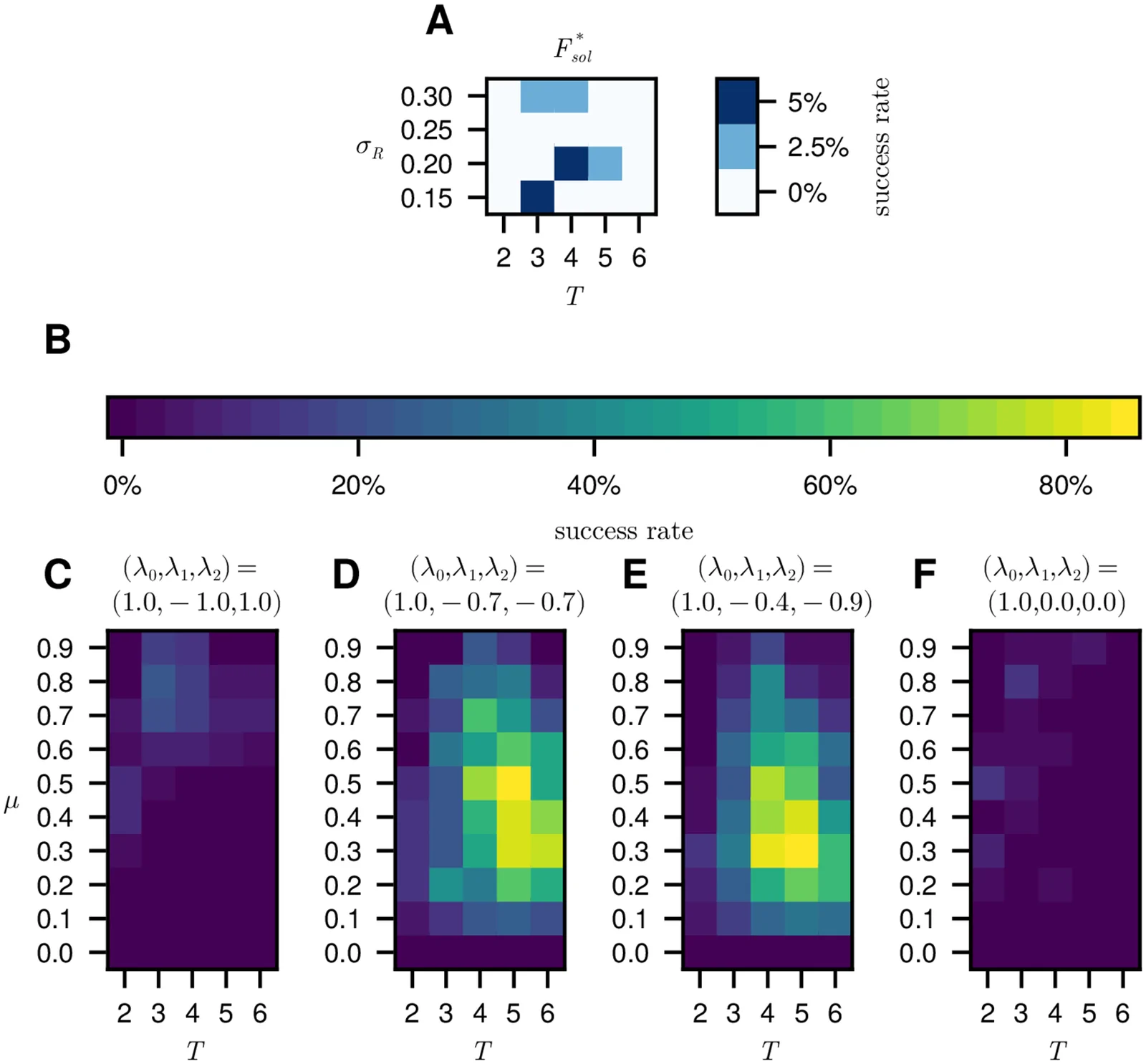
Comparison of simulation setups. Success rates for the pure morphometric approach, i.e., $\left(\lambda_{0},\lambda_{1},\lambda_{2})=(0.0,0.0,0.0\right)$ depending on rotational perturbation parameter $\sigma_{R}$ and simulation temperature *T* (A). A colorbar for the success rate for a mixture $E_{comb}=\mu F_{sol}^{*}+(1-\mu )𝒯$ (B). Heatmaps of the success rate depending on simulation temperature *T* and weighting μ of topological potential 𝒯, for parameters $\left(\lambda_{0},\lambda_{1},\lambda_{2})=(1.0,-1.0,1.0\right)$ (C), $\left(\lambda_{0},\lambda_{1},\lambda_{2})=(1.0,-0.7,-0.7\right)$ (D), $\left(\lambda_{0},\lambda_{1},\lambda_{2})=(1.0,-0.4,-0.9\right)$ (E) and $\left(\lambda_{0},\lambda_{1},\lambda_{2})=(1.0,0.0,0.0\right)$ (F).

All simulations are run with the perturbation parameter $\sigma_{t}=1.25$, which is the standard deviation for the normal distribution from which the translation perturbations are drawn; see Materials and Methods. Fig 4A, shows the success rates of simulations driven by $F_{sol}^{*}$ alone, depending on temperature *T* and rotational perturbation parameter $\sigma_{R}$. Taking the value with the highest aggregated success rate in the temperature range, we set $\sigma_{R}=0.2$ for all simulations driven by different mixtures $E_{comb}$.

### Weight-selection for the topological potential

We select different combinations for triples of $\left(\lambda_{0},\lambda_{1},\lambda_{2}\right)$ defining 𝒯. We pick (1.0,−0.4,−0.9), which corresponds to the point in the parameter space where the minimizer of 𝒯 for three subunits has touching but non-overlapping protein subunits. We further select (1.0,−0.7,−0.7), which lies in the region of low distance between the correct assembly and the topological potential minimizer for two subunits. We chose (1.0, 0.0, 0.0) to see how much of the impact can be attributed to the topological potential simply pulling the proteins together. And finally we pick (1.0,−1.0,1.0) because it relates the topological potential to the integrated Gaussian curvature as:


$$\begin{array}{c} {𝒯}_{\left(1.0,-1.0,1.0\right)}=\frac{1}{4\pi }\int_{\alpha_{0}}^{\alpha_{n}}X(ℬ(\alpha ))-1\;d\alpha . \end{array}$$

Here, $\alpha_{0}$ and $\alpha_{n}$ are the lowest and highest values for which topological changes occur in the filtration of weighted Alpha complexes and ℬ(α) is the corresponding union of balls. See Materials and Methods for details.

### Scanning the parameter space for TMV dimerization

Fig 4C-4F shows heat maps for the choices of $\left(\lambda_{0},\lambda_{1},\lambda_{2}\right)$ mentioned above, where we alter the simulation temperature *T* = 3,4,5,6 and the factor μ=0.1,0.2,...,0.9 that interpolates between $F_{sol}^{*}$ and 𝒯 to calculate the total energy as $E_{comb}=\mu F_{sol}^{*}+(1-\mu )𝒯$. For each point in the heatmap, we run 40 simulations, where each simulation is run for 50000 iterations. This corresponded to roughly 12 hour long simulations on a single core of a high performance cluster. We report the success rate uncertainty as a Wilson score 95% confidence interval [34], which expresses that given the sample size and success rate, we are 95% confident that its true value lies within this interval. We write the interval in square brackets following the success rate.

For the pure morphometric approach, the best success rate we achieve is 5% [1.4, 16.5]. The highest success rate among mixtures of both the morphometric approach and the topological potential is 85% [70.9, 92.9] for ${𝒯}_{\left(1.0,-0.7,-0.7\right)}$ (Fig 4D) at a simulation temperature of 5.0 and a weighting μ=0.5, as well as for ${𝒯}_{\left(1.0,-0.4,-0.9\right)}$ (Fig 4E) at *T* = 5.0 and μ=0.3. This corresponds to a 16-fold increase in the success rate.

The highest success rate for ${𝒯}_{\left(1.0,-1.0,1.0\right)}$ (Fig 4C) is equal to 22.5% [12.3, 37.5]. It is found for *T* = 3.0 and μ=0.8. Although substantially lower than for the other variants, it is still a 4-fold increase over the pure morphometric approach.

The combination of $F_{sol}^{*}$ with ${𝒯}_{\left(1.0,0.0,0.0\right)}$ (Fig 4F) achieves its best success rate of 12.5% [5.5, 26.1] at *T* = 3.0 and μ=0.8. From this we infer that the improvements in success rate are not obtained only because minimizing total zero-dimensional persistence is pulling the subunits together.

### Analysis of a successful simulation of the combined potential

**Fig 5:**
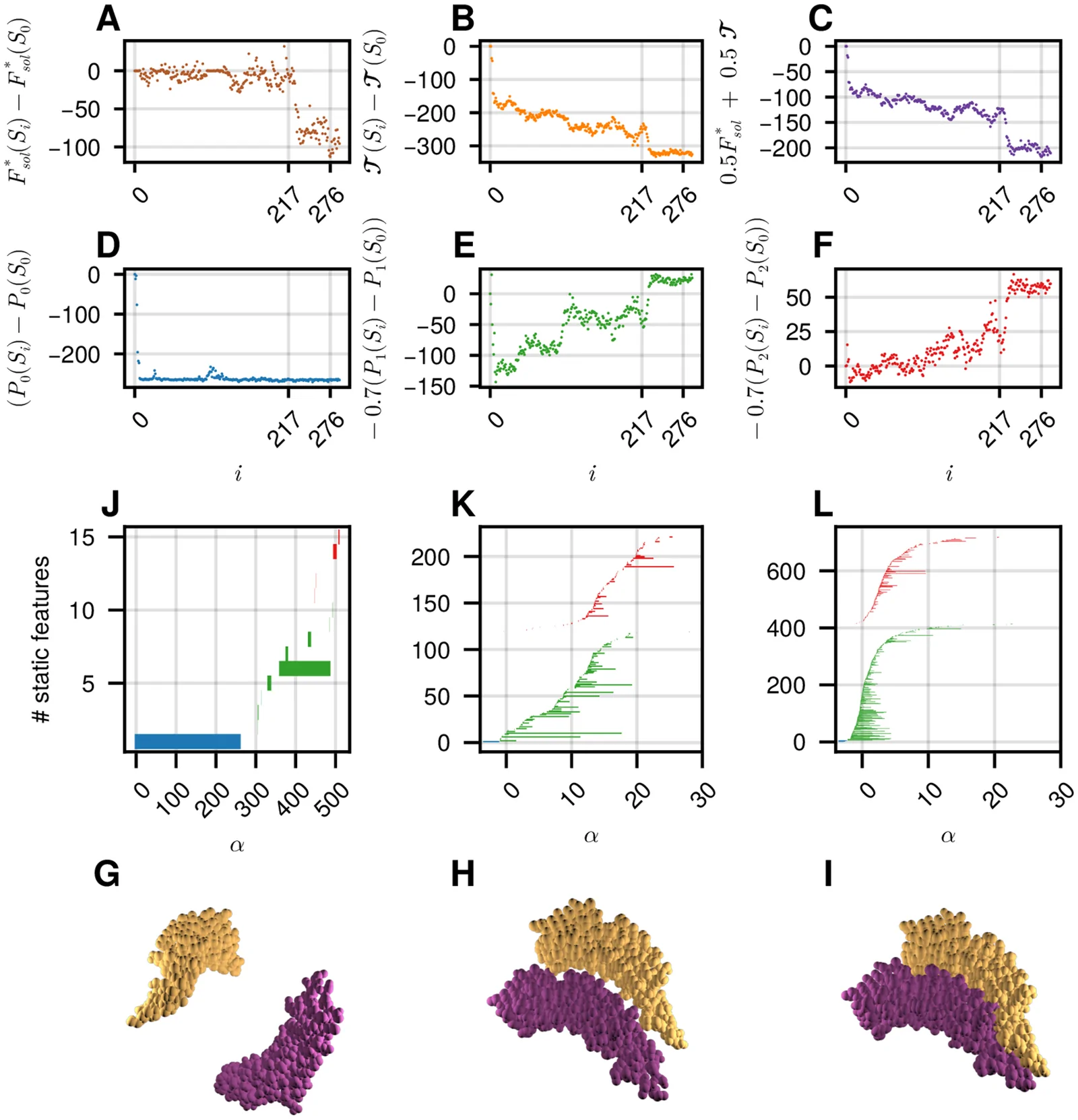
Analysis of final steps. Analysis of the final steps of a simulation driven by $E_{comb}=\frac{5}{10}F_{sol}^{*}+\frac{5}{10}{𝒯}_{\left(1.0,-0.7,-0.7\right)}$. All energy plots (A-F) show values relative to step 0. We see $F_{sol}^{*}$ as given by Eq 1 (A), ${𝒯}_{\left(1.0,-0.7,-0.7\right)}$ (B), $E_{comb}$ (C), and the individual persistence measures (D-F). We show persistence bar plots of steps 0, 217 and 276 with zero-dimensional features in blue, one-dimensional features in green and two-dimensional features in red (J-L). The corresponding protein configurations are shown in (G-I).

Fig 5 illustrates a sample simulated assembly pathway of two subunits with a mixture at μ=0.5 and *T* = 2.0 for $𝒯=P_{0}-0.7P_{1}-0.7P_{2}$. The simulation reaches its minimum energy, a correctly assembled configuration, after 276 steps. The figures in subfigures A-C show $F_{sol}^{*}$, 𝒯 and their mixture relative to the initial (dispersed) state. Subfigures D-F show the individual persistence measures. Subfigures J-L show the persistence barplots for three selected configurations: the initial state (*i* = 0), a state in which the subunits are correctly aligned but have a gap between them (*i* = 217) and finally the minimal energy state (*i* = 276), in which the subunits are correctly assembled. Renders of the corresponding configurations are shown in G-I.

Note that $F_{sol}^{*}$ fluctuates around its initial value until step 217, while the topological potential steadily declines. In the beginning most of the decline is driven by the disappearing zero-dimensional hole, but after about 20 iterations the total persistence for dimension zero remains roughly constant, while the total persistence values for *P*_1_ and *P*_2_ decline. Interestingly, *P*_1_ is almost as low as in the beginning as in the end, caused by a single very persistent feature. As the proteins get pulled together its value increases and then steadily declines. The same can be observed for *P*_2_, although much less pronounced. This matches the observation from the phase diagram in Fig 3, where configurations similar to the initial configuration are minimizers in parts of the phase diagram adjacent to the point in the parameter space we chose. After the initial period, where the proteins are pulled together, the decrease in topological potential is mainly driven by *P*_1_ and supported by *P*_2_.

We refer to the sequence of steps before the simulation attains its minimum, but after the solvent-accessible surfaces begin to overlap as the *final phase*.

In the final phase, both the topological and geometric potentials rapidly decline, while the final gap between the protein subunits is closed. Considering just the topological potential, we observe that the final phase accounts for one third of the total decline, whereas the other two thirds occur during topologically driven alignment of the proteins. Looking at the persistence diagrams in subfigures J-L, we observe that more persistence features appear as the subunits get closer to the correct assembly and that the persistence features are born at lower α values and persist for a shorter time period.

### Analyzing the impact of the topological potential in different simulation phases

To quantify the impact of the topological potential at different stages of the assembly process, we compare the performance of $F_{sol}^{*}$ to $E_{comb}=\frac{1}{2}F_{sol}^{*}+\frac{1}{2}{𝒯}_{\left(1.0,-0.7,-0.7\right)}$ in a set of simulations that are initialized as the first state in the final phase of successful simulation runs. We take 20 such states for both potentials, which are closest in $d_{RMSD}$ to the correct assembly. We then start ten simulations from each of the 40 states for both potentials. Each simulation proposes 10000 steps. The following Table 2 shows the success rates for different combinations of initial states ($F_{sol}^{*}$ touch and $E_{comb}$ touch) and the energies driving the assembly ($F_{sol}^{*}$ finish and $E_{comb}$ finish). We compare the values of the combined potential at two different temperature values to verify the impact of potential detachment and realignment of the subunits.

**Table 2:** Comparing success rates. Comparing success rates with confidence intervals of $\begin{array}{c} F_{sol}^{*} \end{array}$ and $\begin{array}{c} E_{comb} \end{array}$ on first states of final phases of successful simulations for both potentials.

	$F_{sol}^{*}$ touch	$E_{comb}$ touch
$F_{sol}^{*}$ finish, *T* = 3.0	1% [0.3,3.6]	6% [3.5,10.2]
$E_{comb}$ finish, *T* = 3.0	25% [19.5,31.4]	63% [56.1,69.4]
$E_{comb}$ finish, *T* = 5.0	36% [29.7,42.8]	54% [47.1,60.1]

The key takeaway from the table is that the topological potential is useful in *both* stages of the assembly process. That is, during long-distance alignment and short-range docking. $F_{sol}^{*}$ simulations initialized on states taken from successful $F_{sol}^{*}$ simulations find the correct assembly again in 1% of the cases. $F_{sol}^{*}$ simulations initialized on states taken from successful $E_{comb}$ simulations correctly assemble in 6% of the cases. This implies that alignment by $E_{comb}$ is better than alignment by $F_{sol}^{*}$.

We simulate $E_{comb}$ for both *T* = 3.0 and *T* = 5.0 and see that in both cases simulations are more likely to succeed on the set of states that were aligned by $E_{comb}$. Interestingly, the lower temperature performs better in the $E_{comb}$ aligned states. We assume that this is caused by the selected states being comparatively good and that a higher temperature yields a higher chance to move away from them than a lower temperature. What is a drawback for well-aligned states is a feature for the low-quality alignments that appear during pure $F_{sol}^{*}$ simulations. Here, the higher temperature performs better, because moving away from the initialized alignment and realignment driven by $E_{comb}$ is favorable.

Overall, we conclude that the topological potential is a useful long-range augmentation of the morphometric approach because it draws the proteins together in a way that assists assembly and because it further supports assembly at close range.

### Improvements for other systems

**Fig 6:**
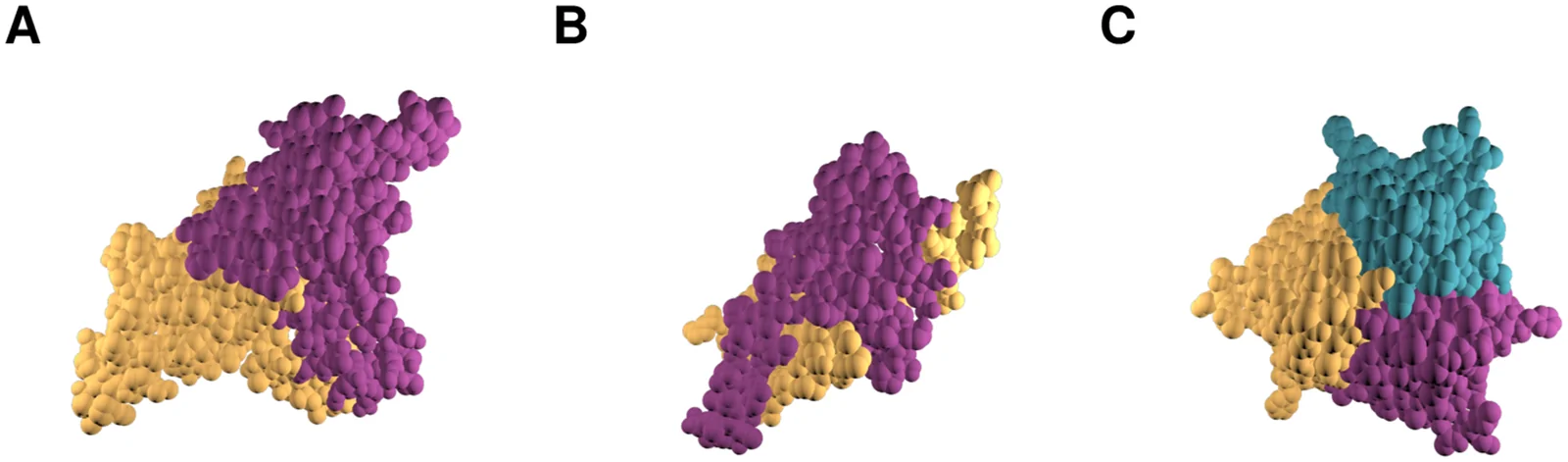
Simulated protein assemblies. Different simulated protein assemblies. Hepatitis B virus core protein (A), SARS-CoV-2 ORF9b (B) and fragment of the Human CD40 Ligand (C). Subunit geometry is given by the A-chains as specified in protein data base entries 4BMG, 6Z4U and 1ALY respectively.

To show that this approach can be generalized, we apply it to simulate dimerization of the human hepatitis B virus core protein [35], dimerization of an accessory protein of the SARS-CoV-2 virus that hinders the immune response [36], and trimerization of an extracellular fragment of the human CD40 ligand, relevant to T cell function [37]. All of these structures are assemblies of homodimers and homotrimers, respectively, which means that they are composed of two or three identical protein chains. Fig 6 shows the renderings of the final assembled states found via simulations driven by


$$\begin{array}{c} E_{comb}=\frac{3}{10}F_{sol}^{*}+\frac{7}{10}{𝒯}_{\left(1.0,-0.4,-0.9\right)}. \end{array}$$

The simulation results are at a distance to the experimental assemblies of $d_{\text{RMSD}}\approx 1\;Å$. The rendered assemblies are therefore visually indistinguishable from the experimentally determined structures, which is why we do not show the latter separately.

For systems of two subunits we run simulations for the pure morphometric approach at T∈{2.0,3.0,4.0}; for combinations with ${𝒯}_{\left(1.0,-1.0,1.0\right)}$ at μ=0.8 and T∈{2.0,3.0,4.0}; and for combinations with ${𝒯}_{\left(1.0,-0.4,-0.9\right)}$ at μ=0.3 and T∈{4.0,5.0,6.0}. For the system with three subunits, that is, the human CD40 ligand, we run simulations at *T* = 5.0. For each combination of parameters we run 40 simulations with 50000 iterations for two subunits and 90000 iterations for three subunits.

Table 3 shows the highest success rate found for any temperature and the potentials described above. We include the previously discussed values of TMV dimerization for comparison.

**Table 3:** Success rates across diverse protein systems. The success rate (sr) of simulations driven by the combined geometric-topological potential ($\begin{array}{c} E_{comb} \end{array}$) is compared against the purely geometric potential ($\begin{array}{c} F_{sol}^{*} \end{array}$) across four diverse protein systems. Wilson score 95% confidence intervals are given in brackets.

Protein	Atoms per	sr [Wilson CI]	sr [Wilson CI]	sr [Wilson CI]
System	subunit	$F_{sol}^{*}$	$\frac{8}{10}F_{sol}^{*}+\frac{2}{10}{𝒯}_{\left(1.0,-1.0,1.0\right)}$	$\frac{3}{10}F_{sol}^{*}+\frac{7}{10}{𝒯}_{\left(1.0,-0.4,-0.9\right)}$
TMV	1206	5.0% [1.4, 16.5]	22.5% [12.3, 37.5]	85.0% [70.9, 92.9]
Hep. B	1134	0.0% [0.0, 8.8]	17.5% [8.7, 32.0]	80.0% [65.2, 89.5]
ORF9b	639	2.5% [0.4, 12.9]	7.5% [2.6, 19.9]	42.5% [28.5, 57.8]
CD40	1113	0.0% [0.0, 8.8]	0.0% [0.0, 8.8]	5.0% [1.4, 16.5]

The key takeaways from the table are that the combined potentials perform better than the morphometric approach in all cases. In particular, for dimerization of the hepatitis B core and trimer assembly of the human CD40 ligand fragment, we observe an increase in the success rate from 0% for the pure morphometric approach to 80.0% and 5.0% respectively, for the potential given by $\frac{3}{10}F_{sol}^{*}+\frac{7}{10}{𝒯}_{\left(1.0,-0.4,-0.9\right)}$. The success rate of dimerization of the SARS-CoV-2-ORF9b protein increases from 2.5% to 42.5% for the same potential. Judging by the values for the pure morphometric approach, the tobacco mosaic virus subunits appear easier to assemble, probably due to the high geometric complementarity compared to the hepatitis B core protein, while simultaneously not being as intricately interlocked as the SARS-CoV-2-ORF9b protein. An animation of the simulated assembly of a CD40 trimer is included in the supporting information (SI). For all systems except CD40, the confidence interval of the best combined potential lies entirely and substantially above that of the pure morphometric approach, indicating that the improvement is not an artifact of the finite number of runs.

It is notable that the selection of parameters that yielded the best results for the tobacco mosaic virus also yields strong improvements for other systems. The fact that it performs better for the hepatitis B core than the SARS-CoV2-ORF9b protein is likely caused by the existence of other states that are energetically close to correct assembly. Indeed, simulations of the SARS-CoV-2-ORF9b protein with energy $E_{comb}=\frac{3}{10}F_{sol}^{*}+\frac{7}{10}{𝒯}_{\left(1.0,-0.4,-0.9\right)}$ are the only ones in which the lowest energy state found is not the native assembly, but a state close by, with $d_{\text{RMSD}}=4.5Å$. However, the minimum of the pure morphometric approach corresponds to the correct assembly and therefore larger values for μ ensure that correctly assembled configurations are obtained as the minimizer.

In all other cases in which we observed at least one simulation that obtained a minimum corresponding to the biologically correct assembly, this structure is also minimizing $E_{comb}$. As such, the combined potential is thermodynamically sound in the sense that, for sufficiently large μ, it correctly distinguishes the ground truth as the energy minimum.

## Discussion

Our study demonstrates that a topological potential, defined as the weighted sum of total persistence measures of proteins in a solvent, substantially improves the success rate of simulated protein assemblies. Acting as a long-range complement to the short-range morphometric approach to solvation free energy, it enables assembly in systems that fail under the morphometric potential alone and increases success rates by more than an order of magnitude for other systems, reaching 85% for TMV dimers with optimized parameters.

We analyze the relationship between the topological potential 𝒯 and the morphometric solvation term with a soft-sphere constraint $F_{sol}^{*}$ in depth for the assembly of TMV dimers. Parameters optimized for TMV translate well to other systems, particularly those with protein subunits of comparable atomic size, yielding high success rates without system-specific tuning.

While the topological potential does not yet have a physical interpretation, one specific parameterization, using the alternating sum of total persistence measures, can be mathematically linked to the Gaussian curvature term in the morphometric approach. This may be understood as capturing topological changes in an expanding molecular surface, potentially connected to solvation shell organization or other long-range, shape-dependent effects.

In practice, the computational cost of calculating the geometric and topological potentials scales linearly in the number of protein subunits, or equivalently the number of atoms. The bottleneck for assembling larger systems is therefore not the cost of a single potential evaluation, but the number of iterations required to reach an assembled configuration. As the configuration space grows exponentially with the number of subunits, this number of iterations increases rapidly with system size; we observed such a rapid increase already for the purely short-ranged morphometric approach [27]. The improved efficiency observed when integrating the topological potential as a simulation strategy facilitates the assembly of larger systems. Coarse-graining [38] by reducing the number of modeled atoms per subunit is a feasible approach to increase the accessible system size further, as long as sufficient shape-specificity is retained that allows for the alignment of geometric locks and keys. In general, improvements in the running time of the topological potential, through coarse-graining, optimization or parallelization, would make it feasible to simulate flexible molecules and larger assemblies of tens to hundreds of subunits.

While all systems studied here are homoassemblies of at most three subunits, the method does not depend conceptually on either restriction, and preliminary work including ligands and proteins shows it extends well.

The calculation of the topological potential 𝒯 only depends on the geometry of the atoms and the choice for the parameters $\lambda_{i}$. Its integration into a simulation-governing energy function offers a general computational strategy to overcome rugged energy landscapes in shape assembly simulations. Its performance is demonstrated here across diverse protein systems. The method recovers the thermodynamically accurate states observed in nature with high probability given enough iterations to scan the configuration space. Hence it can be utilized to find low energy states of arbitrary, in particular unknown, molecular assemblies. It retains physical realism in the form of thermodynamic accuracy, but does not model dynamics like molecular dynamics (MD) simulations and therefore does not allow for the inference of kinetic accessibility. However, similarly to MD methods the approach does not depend on prior knowledge of the target states similar to the modeled system. This is in contrast to deep-learning methods like AlphaFold3, whose accuracy on docking tasks has recently been shown to depend strongly on the similarity of the target to the training set [39]. While the mechanism by which the topological potential guides assembly is not yet fully understood, the potential itself is transparent: it is a weighted sum of the total persistence of a weighted alpha complex, a visualizable geometric construction governed by atom geometry and three parameters $\lambda_{i}$. In this sense it is not a black box in the way a deep neural network is, whose behavior is determined by billions of parameters and is correspondingly difficult to interpret. The topological potential by itself can be viewed as a long-range shape-alignment and matching potential, making it useful for simulations in computational biology and materials science.

## Materials and methods

### Parameters for the morphometric approach

For a hard-sphere solvent the physical prefactors for the four terms in the morphometric solvation free energy can be computed using the White Bear functional Mark II [26]. Each term is given as a function depending on the packing fraction η and the solvent radius $r_{s}$:


$$\begin{array}{c} \beta p=\frac{\eta }{r_{s}^{3}}\frac{3}{4\pi }\left(\frac{1+\eta +\eta^{2}-\eta^{3}}{(1-\eta {)}^{3}}\right), \end{array}$$

$$\begin{array}{c} \beta \sigma =\frac{\eta }{r_{s}^{2}}\frac{3}{4\pi }\left(-\frac{1+2\eta +8\eta^{2}-5\eta^{3}}{3(1-\eta {)}^{3}}-\frac{\ln(1-\eta )}{3\eta }\right), \end{array}$$

$$\begin{array}{c} \beta \kappa =\frac{\eta }{r_{s}}\frac{3}{4\pi }\left(\frac{4-10\eta +20\eta^{2}-8\eta^{3}}{3(1-\eta {)}^{3}}+\frac{4\ln(1-\eta )}{3\eta }\right), \end{array}$$

$$\begin{array}{c} \beta \overset{―}{\kappa }=\eta \frac{3}{4\pi }\left(\frac{-4+11\eta -13\eta^{2}+4\eta^{3}}{3(1-\eta {)}^{3}}-\frac{4\ln(1-\eta )}{3\eta }\right). \end{array}$$

Here β is the inverse thermodynamic temperature, which we set to 1.0*J*^-1^, since we are only interested in relative energy levels. Mimicking the properties of water we set $r_{s}=1.4Å$ and η=0.3665 (the approximate fluid density relevant to biological processes). This yields the values of $p\approx 0.182\frac{J}{{Å}^{3}}$, $\sigma \approx -0.131\frac{J}{{Å}^{2}}$, $\kappa \approx 0.112\frac{J}{Å}$ and $\overset{―}{\kappa }\approx -0.03J$.

In Equation 1 we augment the morphometric approach by a linear overlap penalty *L* to energetically penalize overlapping protein subunits as well as to relax the rigidity of the shapes. The weighting ϕ for *L* is determined empirically [27].

### Persistent homology of Alpha filtrations

The geometric and topological measures are derived from the weighted Alpha complex of the atomic centers. The Alpha complex is a subcomplex of the weighted Delaunay triangulation that provides a mathematically precise and computationally efficient representation of the union-of-balls model of a molecule [29,40]. For a set of atomic centers $P=\{p_{1},...,p_{n}\}\subset {\mathbb{R}}^{d}$ and a corresponding set of weights $W=\{w_{1},...,w_{n}\}\subset \mathbb{R}$, the construction begins with the *weighted Voronoi diagram*. The *i-th weighted Voronoi cell* is defined as


$$\begin{array}{c} V(p_{i},w_{i}):=\{x\in {\mathbb{R}}^{d}|\pi_{p_{i},w_{i}}(x)\leq \pi_{p_{j},w_{j}}(x),\forall j\in \{1,...,n\}\}, \end{array}$$

where $\pi_{p,w}(x)=||x-p|{|}^{2}-w$ is the power distance. The collection of weighted Voronoi cells is the weighted Voronoi diagram denoted by 𝒱(P,W).

Under the assumption that no *d* + 2 Voronoi cells intersect in a common point, i.e., the points in *P* are in general position, the dual to the weighted Voronoi diagram is the *weighted Delaunay triangulation*.

Now let $W=\{w_{1},...,w_{n}\}\subset \mathbb{R}$ be a set of weights assigned to the points in *P*, where each weight $w_{i}$ is the squared atomic radius ($w_{i}=r_{i}^{2}$). Let $B(p_{i},\sqrt{w_{i}})$ be the ball centered at point $p_{i}$ with radius $r_{i}=\sqrt{w_{i}}$. We denote


$$\begin{array}{c} R(p_{i},w_{i})=V(p_{i},w_{i})\cap B(p_{i},\sqrt{w_{i}}). \end{array}$$

The *weighted Alpha complex* is defined as


$$\begin{array}{c} 𝒜(P,W)=\{\sigma \subseteq P|\underset{p_{i}\in \sigma }{⋂}R(p_{i},w_{i})\text{?}\emptyset \}, \end{array}$$

i.e., the dual of the weighted Voronoi cells intersected with the balls $B(p_{i},\sqrt{w_{i}})$. Inclusion-Exclusion formulas to compute the geometric measures *V*, *A*, *C* and *X*, are based on this incidence information [40–44]. These formulas are implemented in the AlphaMol program [32]. We also use the weighted Alpha complex to compute the overlap penalty *L*, by iterating over the edges whose vertices correspond to different proteins and summing up the overlap of the corresponding balls.

Let α∈ℝ. We define $B(p_{i},w_{i},\alpha )$ as the ball centered at $p_{i}$ with radius $r_{i}=\sqrt{w_{i}+\alpha }$, where $B(p_{i},w_{i},\alpha )=\emptyset$ if $\alpha <-w_{i}$. We get that for points *x* with equal power distance to balls *i* and *j*:


$$\begin{array}{c} \pi_{p_{i},r_{i}^{2}}(x)=||x-p_{i}|{|}^{2}-(w_{i}+\alpha )=||x-p_{j}|{|}^{2}-(w_{j}+\alpha )=\pi_{p_{j},r_{j}^{2}}(x), \end{array}$$

which is equivalent to $||x-p_{i}|{|}^{2}-w_{i}=||x-p_{j}|{|}^{2}-w_{j}$ as the α cancels out. Therefore, the weighted Voronoi diagram is the same for all values of α. We write


$$\begin{array}{c} W_{\alpha }=\{w+\alpha |w\in W\} \end{array}$$

and get a nested sequence of complexes


$$\begin{array}{c} 𝒜(P,W_{\alpha_{0}})\subset ...\subset 𝒜(P,W_{\alpha_{m}}) \end{array}$$

for critical values of alpha $\alpha_{0}\leq ...\leq \alpha_{m}$. The critical values are the lowest values for which a simplex in the weighted Delaunay Triangulation appears in the corresponding weighted Alpha complex.

Given a filtration $F={𝒜}_{0}\subset ...\subset {𝒜}_{m}$ and homomorphisms, induced by the inclusion maps, $f_{p}^{i,j}:H_{p}({𝒜}_{i})\to H_{p}({𝒜}_{j})$, the images


$$\begin{array}{c} H_{p}^{i,j}=\mathrm{im}(f_{p}^{i,j}) \end{array}$$

are the *p*-dimensional *persistent homology groups*. Their ranks


$$\begin{array}{c} \beta_{p}^{i,j}=\mathrm{rank}(H_{p}^{i,j}), \end{array}$$

are the *p*-dimensional *persistent Betti numbers*. They count the *p*-dimensional cycles that are not boundaries. Given a class $\gamma \in H_{p}({𝒜}_{i})$ we say γ is *born* at ${𝒜}_{i}$ if $\gamma \notin H_{p}^{i-1,i}$. We say it *dies* at ${𝒜}_{j}$ if $f_{p}^{i,j-1}(\gamma )\notin H_{p}^{i-1,j-1}$ and $f_{p}^{i,j}(\gamma )\in H_{p}^{i-1,j}$. We call the interval $\left(\alpha_{i},\alpha_{j}\right)$ the *birth-death interval* of γ and $\alpha_{j}-\alpha_{i}$ its *persistence*. We refer to the collection of all *p*-dimensional birth-death intervals as the *p*-dimensional *persistence diagram* and denote it by $Dgm_{p}$ [45].

We use the Oineus software package [46] to calculate persistent homology.

### Simulation methods and parameters

We simulate configurations of protein subunits using variants of a Random Walk Metropolis [47]. Each configuration is represented by a state $x\in ({\mathrm{SO}}_{3}\times {\mathbb{R}}^{3}{)}^{m}$, where *m* is the number of simulated molecules. The geometries of the systems we study are taken from the protein data base entries with identifiers 6R7M [48], 4BMG [35], 6Z4U [36] and 1ALY [37], for TMV, hepatitis B, SARS-CoV2 and the CD40 ligand respectively. For atomic radii we use the *ProtOr* radii [49], which account for hydrogen atoms by giving radii for atomic groups instead of individual atoms. Each simulation is run in a bounding box of size 120Å. These bounds are not checked for each individual atom but for each center of mass of the simulated molecules.

A perturbation for a molecule consists of a translation and a rotation. The translational perturbation vector is generated by drawing three independent samples from a normal distribution $𝒩(0,\sigma_{t}^{2})$ with $\sigma_{t}=1.25Å$, and this vector is added to the molecule’s current position. The rotational perturbation is generated by first creating a three dimensional random vector *v*, whose components are also drawn independently from a normal distribution, $𝒩(0,\sigma_{R}^{2})$, with $\sigma_{R}=0.2$. This vector *v* represents an element of the Lie algebra so(3). It is then mapped to a rotation in SO(3) via the exponential map. This resulting small, random rotation is then composed with the molecule’s existing orientation to yield the new candidate orientation.

Assuming the current state of the simulation is state $x^{i}$, we pick one of the simulated molecules at random and perturb it to generate a candidate state $x^{\prime }$. This is also known as *component-wise* or *block-wise* Random Walk Metropolis [50].

The probability of accepting $x^{\prime }$ as the new state of the simulation is determined as:


$$\begin{array}{c} \min\left(1,\exp\left(\frac{-\Delta E}{T}\right)\right), \end{array}$$

where $\Delta E=E(x^{\prime })-E(x^{i})$ is the difference in energy of the states. If $x^{\prime }$ is accepted, we set $x^{i+1}=x^{\prime }$. Otherwise we set $x^{i+1}=x^{i}$. *T* is a simulation temperature, which regulates the probability of accepting states with higher energy. The energy used here is $E_{comb}$ as in Eq 3 for different parameters μ and $\lambda_{i}$. The prefactors of $F_{sol}^{*}$ are given by the White Bear prefactors [26] computed for packing fraction η=0.3665 and solvent radius $r_{s}=1.4Å$, mimicking the properties of water. For the simulated annealing (SA) [51] simulations we change *T* over the course of a simulation by letting it decay to zero according to a quadratic annealing schedule:


$$\begin{array}{c} T_{k}=T_{0}{\left(\frac{I-k}{I}\right)}^{2}, \end{array}$$

where *I* is the total number of iterations at which the temperature reaches zero and *k* is the current step in the decay period. Once *T* = 0.0 is reached, we reheat the system several times to scan for nearby local minima.

In cases in which we did not know which initial temperature to start a simulation with, we run 12 shorter RWM simulations and check whether the total acceptance rate is above or below some target acceptance rate. If it is below the target acceptance rate, we multiply the initial temperature for the next simulation by a factor of 1.5, if it is above the target acceptance rate we multiply the initial temperature for the next simulation by a factor of 0.5. After the 12 simulations are finished we pick the initial temperature closest to our target acceptance rate. For RWM simulations we choose a target acceptance rate of 0.2 and for SA simulations we choose a target acceptance rate of 0.8.

The morphometric approach is short ranged and additive. As such it is not necessary to recompute its energetic contribution of the whole system in every step, but only the connected components of molecules that have changed. We determine the connected components in each step by constructing a graph that has each molecule as a node and an edge whenever the bounding spheres of two molecules overlap. Let *idx* be the index of the molecule we moved for the proposal of simulation step *i* + 1. Let ${𝒞}_{i}$ be the set of connected components from step *i* and let ${𝒞}_{i+1}$ be the set of connected components from step *i* + 1. For each component $c\in {𝒞}_{i+1}$ there are three cases to consider. Case 1: The connected component *c* is an element of ${𝒞}_{i}$ and idx∉c. Then the energy of *c* remains unchanged and we can just copy the energy value from the last iteration. Case 2: The connected component *c* is an element of ${𝒞}_{i}$ and idx∈c. Then the energy of *c* changes and we have to recompute it. Case 3: The connected component *c* is not an element of ${𝒞}_{i}$. Then the energy of *c* is changed and we need to recompute. By passing the energy value of a single molecule to the algorithm we can further optimize this procedure and not recalculate the energetic contributions of components that consist of only one molecule.

To ensure a fair comparison between different topological potential setups, we defined a relative contribution measurement, $c_{𝒯}$. This measurement quantifies the energetic impact of the topological term relative to the geometric term during the last stage of the assembly process. We take the set of 800 simulations, the data shown in Fig 4A are based on. For every simulation *i* we take two states $S_{min}^{i}$, the minimal energy state, and $S_{disp}^{i}$, the state immediately before the solvent-accessible surface areas of the protein subunits start to interact, before $S_{min}^{i}$ is reached. We then calculate an average of how much of the change in energy in the final phase in these simulations is contributed by 𝒯 in relation to $F_{sol}^{*}$ as:


$$\begin{array}{c} c_{𝒯}=\frac{1}{800}\sum_{i=1}^{800}\frac{𝒯(S_{min}^{i})-𝒯(S_{disp}^{i})}{F_{sol}^{*}(S_{min}^{i})-F_{sol}^{*}(S_{disp}^{i})}. \end{array}$$

We find that $c_{{𝒯}_{\left(1.0,-0.7,-0.7\right)}}\approx 1.12$, $c_{{𝒯}_{\left(1.0,-0.4,-0.9\right)}}\approx 0.96$ and $c_{{𝒯}_{\left(1.0,-1.0,1.0\right)}}\approx 0.61$, i.e., the values are reasonably similar for the different setups, ensuring comparability. Note that calculating $c_{{𝒯}_{\left(1.0,0.0,0.0\right)}}$ is not very informative because zero dimensional persistence has little impact in the final stage of a simulation.

### Relation between the topological potential and the integrated Gaussian curvature

Consider a set of atom centers $P=\{p_{1},...,p_{n}\}$ and van der Waals radii $R=\{r_{1},...,r_{n}\}$. The set of weights


$$\begin{array}{c} W=\{(r_{j}+r_{s}{)}^{2}|r_{j}\in R\} \end{array}$$

for the solvent radius $r_{s}$ are the weights of the weighted Alpha complex used to compute the morphometric measures. Now let $ℬ(\alpha )=\overset{m}{\underset{j=1}{⋃}}B_{j}(\alpha )$ where $B_{j}(\alpha )$ is the ball centered at $p_{j}$ with radius $\sqrt{w_{j}+\alpha }$. If $w_{j}+\alpha <0$, we define $B_{j}(\alpha )=\emptyset$. Because the Alpha complex ${𝒜}_{\alpha }$ is homotopy equivalent to the union of balls ℬ(α), their Euler characteristics are equal [40]. The sequence of values, $\alpha_{0}<...<\alpha_{k}$ for which topological changes occur, when moving from $𝒜(\alpha_{i})$ to $𝒜(\alpha_{i+1})$, are the filtration values of the Alpha filtration on which we calculate persistent homology. Here


$$\begin{array}{c} \alpha_{0}=-\max_{j\in 1,...,m}w_{j}. \end{array}$$

The Euler characteristic χ of a union of balls ℬ(α) is related to its integrated Gaussian curvature *X* (integrated over the boundary) by a factor of 4π. That is:


$$\begin{array}{c} X(ℬ(\alpha ))=4\pi \chi (ℬ(\alpha )). \end{array}$$

Then by the Euler-Poincaré formula [45] we get:


$$\begin{array}{c} \sum_{i=0}^{2}(-1{)}^{i}\sum_{(b,d)\in Dgm_{i}}(d-b)=\int_{\alpha_{0}}^{\alpha_{n}}\chi (ℬ(\alpha ))-1\;d\alpha . \end{array}$$

That is, the alternating sum of total persistence per dimension is equal to the integral of $\alpha_{0}$ to $\alpha_{n}$ over the Euler characteristic χ(ℬ(α)). We subtract 1 in the integral because the connected component representing the whole molecule persists forever, and we do not count it in the alternating sum of the total persistence values.

Therefore:


$$\begin{array}{c} {𝒯}_{\left(1.0,-1.0,1.0\right)}=\int_{\alpha_{0}}^{\alpha_{n}}\frac{1}{4\pi }X(ℬ(\alpha ))-1\;d\alpha . \end{array}$$

This choice of parameters can thus be seen as a range extension of one of the terms of the morphometric approach in a mathematically rigorous sense.

We note that for α≤0, we expect the integral to be roughly constant across different configurations, since the atom centers within a protein subunit are usually closer together than those between subunits. For $\lambda_{0}^{*}=\lambda_{0}-1$, $\lambda_{1}^{*}=\lambda_{1}+1$ and $\lambda_{2}^{*}=\lambda_{2}-1$ we have that:


$$\begin{array}{c} \sum_{i=0}^{2}\lambda_{i}\sum_{(b,d)\in Dgm_{i}}(d-b)=\sum_{i=0}^{2}((-1{)}^{i}+\lambda_{i}^{*})\sum_{(b,d)\in Dgm_{i}}(d-b), \end{array}$$

meaning the topological potential 𝒯, can always be decomposed into:


$$\begin{array}{c} {𝒯}_{\left(\lambda_{0},\lambda_{1},\lambda_{2}\right)}=\int_{\alpha_{0}}^{\alpha_{n}}\frac{1}{4\pi }X(ℬ(\alpha ))-1\;d\alpha +{𝒯}_{\left(\lambda_{0}^{*},\lambda_{1}^{*},\lambda_{2}^{*}\right)} \end{array}$$

Hence, while the morphometric approach has the integrated Gaussian curvature X(ℬ(0)) as a component, the topological potential contains the integral over the Euler characteristic of ℬ(α) over the range of α values in which topological changes occur in the Alpha filtration.

### Ground truths from multiple A-chains

The data provided in the protein data base files 4BMG, 6Z4U and 1ALY contain geometric information for the protein assemblies discussed in Fig 6 and Table 3. The dimers or trimers are assemblies of several proteins of the same type, which have subtle differences in the geometry of each chain due to the non-rigid nature of the proteins or measurement imprecision. We take the A-chain of all entries as the geometric template in the simulations we run. The ground truth to which we compare simulation results is obtained by superimposing the A-chain onto the positions and orientations of the other chains of the dimer or trimer.

### Simulation evaluation

Let $Q,U,V,W\subset {\mathbb{R}}^{3k}$ be rigid transformations of some underlying template $X_{0}\subset {\mathbb{R}}^{3k}$ representing a molecule with *k* atoms.

Now assume that *Q* and *U* are a pair of molecules in some configuration 𝒳 and that *V* and *W* are a pair of molecules in some configuration 𝒴. Let $\left(R_{Q\to V},t_{Q\to V}\right)$ be the optimal rigid-body transformation that superimposes molecule *Q* onto molecule *V*. This transformation is found by solving the minimization problem:


$$\begin{array}{c} (R_{Q\to V},t_{Q\to V})=\underset{R\in SO(3),t\in {\mathbb{R}}^{3}}{arg\;min}\sum_{i=1}^{k}{\left\|(Rq_{i}+t)-v_{i}\right\|}^{2}. \end{array}$$

This problem can be solved efficiently using the Kabsch algorithm [52].

Using this transformation, we define a pairwise RMSD, *d*_pair_. This metric quantifies the structural deviation of the molecule, *U*, from its target, *W*, after the transformation $\left(R_{Q\to V},t_{Q\to V}\right)$ has been applied. Because we assume all molecules are perfect rigid-body instances of the same template, the initial alignment of *Q* onto *V* results in zero error. The metric is therefore defined solely by the deviation of the second molecule:


$$\begin{array}{c} d_{\text{pair}}(Q,U,V,W)=\sqrt{\frac{1}{k}\sum_{i=1}^{k}{\left\|(R_{Q\to V}u_{i}+t_{Q\to V})-w_{i}\right\|}^{2}}. \end{array}$$

Note that the sum is now over the *k* atoms of the second molecule only, and the normalization factor is *k*.

To handle the ambiguity of local assignment within the pair, the final pairwise distance, *d*_minpair_, is the minimum of the two possible alignments, *Q* to *V* or *Q* to *W*:


$$\begin{array}{c} d_{\text{minpair}}(Q,U,V,W)=\min\left(d_{\text{pair}}(Q,U,V,W),d_{\text{pair}}(Q,U,W,V)\right). \end{array}$$

Finally, the total distance *d*_RMSD_ between two configurations, $𝒳=\{X_{1},...,X_{m}\}$ and $𝒴=\{Y_{1},...,Y_{m}\}$, is the average of these pairwise distances, minimized over permutations $\pi \in S_{m}$:


dRMSD(𝒳,𝒴)=minπ∈Sm1(m2)∑1≤i<j≤mdminpair(Xi,Xj,Yπ(i),Yπ(j)),

where the sum goes over all unique pairs of molecules in the configuration.

Intuitively, *d*_RMSD_ finds the optimal global matching of molecules. For each matched pair, it determines the best alignment based on one of the molecules and then measures the resulting RMSD of the entire pair. A *d*_RMSD_ value of zero guarantees that two configurations are identical up to a global rigid transformation.

### Codebase

The code we developed to generate the data presented in this study is available on github [53]. Raw simulation trajectories and analysis scripts are publicly available on Zenodo [54].

## Supporting information

S1 Video Animation of the simulated assembly of a trimer of the human CD40 ligand. (MP4)
