# Supplementary material for: Topological potentials guiding protein self-assembly
Source: PLoS Comput Biol. 2026 Sep 3;22(9):e1014709. doi: 10.1371/journal.pcbi.1014709 (PMC13577540; doi:10.1371/journal.pcbi.1014709)
Supplement: Supplementary file 1 [file pcbi.1014709.s002.pdf]

Review Questions for Topological potentials guiding protein self-assembly

The paper introduces a new computational method to improve simulations of protein self-assembly. The key problem is that existing simulations are driven by a short-range geometric energy function known as morphometric approach. It frequently gets stuck in incorrect configurations because the energy landscape is largely flat at long range but rugged up close. To address this, the authors introduce a long-range "topological potential" derived from persistent homology, a mathematical tool that tracks how the shape of the protein system evolves across multiple length scales. This potential is combined with the existing geometric term into a single energy function, where the topological term acts as a coarse funnel drawing proteins into roughly the right arrangement, and the geometric term handles precise docking once they are close.

The work is well-motivated, clearly written, and the results across four protein systems are compelling. Therefore, the work is suitable for publication in PLOS Computational Biology; however, several issues need to be addressed or discussed before it can be accepted, and minor revisions are recommended.

1. In Figure 2, the x-axis lacks a label
2. Electrostatic interactions is a major driver in biomolecular aggregation. How does the topological and geometrical terms include electrostatics? If not, is it possible to incorporate electrostatics to this model?
3. The repulsive part of van der Waals interactions can be reflected by the excluded volume terms, but how do the attractive van der Waals interactions are captured? If not, which terms/effects attributed to this model lead to attraction between molecules to form molecular assemblies?
4. The overarching utility of this method, as well as reliability and accuracy is not clear in the manuscript. Comparison with experimental observables, and or other methods like atomistic and coarse grained MD simulations is important.
5. In sections where comparisons are made with experimentally determined structures, it would be valuable to include visual snapshots of the experimental structures alongside the model predictions. This would facilitate a more intuitive and direct visual assessment of structural agreement or discrepancy.
6. Although heteroassemblies are acknowledged as a limitation of the current method, it is not clear why they were not tested, even in a preliminary capacity. Given their biological relevance, the authors should provide a more detailed justification for this omission and, if feasible, consider including at least an exploratory analysis or a discussion of the specific challenges involved.
7. In the distance heatmap, the units of distance are not specified, and the color bar lacks appropriate labels. Please add axis units and a clearly labeled color scale to make the figure fully interpretable without reference to the main text.
8. A comparison with state-of-the-art structure prediction tools such as AlphaFold3 would considerably strengthen the manuscript and help contextualize the performance of the proposed method. If a direct computational comparison is not feasible within the scope of this work, the authors should at minimum include a substantive discussion of how their approach relates to, complements, or differs from such tools in terms of methodology, applicability, and expected performance.
