# Supplementary material for: Topological potentials guiding protein self-assembly
Source: PLoS Comput Biol. 2026 Sep 3;22(9):e1014709. doi: 10.1371/journal.pcbi.1014709 (PMC13577540; doi:10.1371/journal.pcbi.1014709)
Supplement: Supplementary file 2 [file pcbi.1014709.s004.pdf]

Response to Reviewers

Topological potentials guiding protein self-assembly

We thank the reviewers for their thorough reviews and the insightful comments they raise. In the following we have attached both reviews, our responses to them and the adjustments we made in the manuscript. We believe that the manuscript has improved substantially as a result of the reviewers' comments and suggestions. Reviewer comments are reproduced in the shaded boxes; our responses follow in blue.

Reviewer #1

The authors study protein self-assembly using a computational approach that combines a morphometric geometric solvation model with a long-range topological potential derived from persistent homology. The central idea is to use weighted total persistence as an energetic bias to guide assembly pathways and reduce kinetic trapping in rugged energy landscapes. The authors apply the approach to several systems, including TMV dimers, hepatitis B core protein, SARS-CoV-2 ORF9b, and CD40 ligand, and report substantial improvements in assembly success rates compared to the geometric model alone.

The manuscript presents an interesting and potentially useful approach for improving simplified assembly simulations. The reported increases in assembly success rates are substantial, and the paper is generally clearly written. However, several points need to be addressed before publication.

1. Physical interpretation of the topological potential

The physical interpretation of the topological potential remains unclear. While the authors briefly relate a specific parameter choice to Gaussian curvature, the manuscript does not provide clear physical intuition regarding whether the topological term represents an effective approximation of omitted long-range interactions (e.g., electrostatic or hydrophobic effects) or functions primarily as an algorithmic bias that reshapes the energy landscape to reduce kinetic trapping. Relatedly, the manuscript frames protein assembly simulations as being dominated by short-range nonpolar interactions, whereas many assembly processes are strongly influenced by electrostatics, hydrogen bonding, and other longer-range effects. Since electrostatic interactions are omitted entirely, it remains unclear how the proposed topological potential compares to more conventional implicit-solvent or coarse-grained models that include electrostatics, and whether the topological term provides fundamentally new information or instead compensates for omitted physical interactions.

Response: We agree with the reviewer that the manuscript should make it more obvious that protein self-assembly is not only driven by short-range interactions. The goal of the simulation was to model the non-polar solvent-driven part of the self-assembly process, which is modeled using the morphometric approach. The topological potential we introduce is a simulation strategy that acts as a long-range shape matching potential. The exact physical interpretation of

this potential is not clear, but from the perspective of shape matching, it behaves in a similar way to long-range physical interactions. We add the following paragraph to the introduction to clarify the raised points:

> *In physical systems long-range interactions such as electrostatics [16] and long-range hydration forces [28] pull the proteins together and correctly align them. The sum of these effects can be interpreted as a long-range shape matching effect. The biasing potential we introduce in the following does not have a direct physical interpretation but acts as a long-range shape matching potential and in this sense is a similar mechanism to long-range physical interactions.*

2. Extension to larger-scale assemblies

> The manuscript focuses on relatively small homo-oligomeric systems, primarily dimers and trimers, rather than full viral capsids or larger supramolecular assemblies. Thus, the reported increases in "assembly success rates" correspond to improved formation of small native-like oligomeric complexes rather than full virus assembly. It would be useful for the authors to discuss more explicitly whether the proposed topological potential is expected to extend to larger-scale coarse-grained assembly simulations involving tens to hundreds of subunits, such as HBV or TMV capsid assembly. In particular, it remains unclear whether persistent homology can be computed efficiently using coarse-grained capsomers or bead-based representations rather than atomistic coordinates, whether the topological potential is expected to guide global shell closure or suppress malformed intermediates during capsid assembly, and how the computational cost is expected to scale with increasing system size.

Response: We answer the remark together with the following comment on rigid-body treatment and conformational flexibility.

3. Rigid-body treatment and conformational flexibility

> Relatedly, the present model treats protein subunits as rigid bodies. In contrast, conformational flexibility often plays an important role in viral assembly pathways and polymorphism, including in coarse-grained models capable of assembling complete viral shells. See, for example, "From Disorder to Icosahedral Symmetry: How Conformation-Switching Subunits Enable RNA Virus Assembly," Science Advances 11, eady7241 (2025). The authors should discuss whether the proposed topological framework could eventually be combined with flexible coarse-grained models and whether it is intended as a complementary guiding potential for improving existing coarse-grained assembly simulations rather than as a standalone assembly model.

Response: The reviewer raises important points. The topological potential is agnostic to the number of subunits and their conformations. It can thus, in principle, be applied to larger assemblies or simulations in which flexible molecules are modeled. However, while the computational cost of calculating the topological potential scales, in practice, linearly in the number of atoms, the number of iterations needed to assemble larger numbers of molecules grows exponentially. As such, the assembly of tens or hundreds of subunits is currently computationally infeasible. The reviewer's idea to run the simulation on a coarse-grained representation of the molecules is a feasible and promising option as long as sufficient shape-specificity is retained that allows for the alignment of geometric locks and keys.
We add the following paragraph to the discussion section of the manuscript:

In practice, the computational cost of calculating the geometric and topological potentials scales linearly in the number of protein subunits, or equivalently the number of atoms. The bottleneck for assembling larger systems is therefore not the cost of a single potential evaluation, but the number of iterations required to reach an assembled configuration. As the configuration space grows exponentially with the number of subunits, this number of iterations increases rapidly with system size; we observed such a rapid increase already for the purely short-ranged morphometric approach [27]. The improved efficiency observed when integrating the topological potential as a simulation strategy facilitates the assembly of larger systems. Coarse-graining [38] by reducing the number of modeled atoms per subunit is a feasible approach to increase the accessible system size further, as long as sufficient shape-specificity is retained that allows for the alignment of geometric locks and keys. In general, improvements in the running time of the topological potential, through coarse-graining, optimization or parallelization, would make it feasible to simulate flexible molecules and larger assemblies of tens to hundreds of subunits.

4. Selection of weighting coefficients and morphometric parameters

The topological potential depends on weighting coefficients $(\lambda_0, \lambda_1, \lambda_2)$, which appear to be selected empirically through parameter scans, but the procedure used to choose these parameters remains somewhat unclear. The authors should clarify how the λ_i values are selected, whether they are optimized systematically or motivated by physical considerations, and how sensitive the results are to these choices. The apparent transferability across systems is interesting, but a more systematic robustness analysis would improve the conclusions.

Similarly, the implementation of Eq. (1) remains somewhat vague. The manuscript should more clearly state the numerical values used for the morphometric parameters, how these parameters are selected, and whether any of them are adjusted during the simulations. This clarification is particularly important for readers unfamiliar with morphometric approaches.

Response: The λ_i are chosen by a grid search. The initial boundaries of the grid are chosen based on observations about how the total persistence values behave when tracking existing simulations. This resulted in the idea to minimize λ_0, while maximizing λ_1 and λ_2. The grid was then extended until the most promising region of the scan lay away from the boundaries. To clarify this, and Eq. 1, we add the following paragraphs to the results and a subsection to the Materials and Methods section:

We calculate the prefactors utilizing the White Bear functional mark II [26] from density functional theory, giving us values for a hard-sphere solvent depending on solvent radius r_s and packing fraction η, which we set to $1.4\,\text{Å}$ and 0.3665 respectively to mimic the properties of water.

In the results section we determine promising candidates for the λ_i based on a grid search in which we explore for which parameters the topological potential alone obtains minima that are energetically close to true assemblies of TMV protein subunits. The initial boundaries of the grid are chosen based on observations about the total persistence measures as well as theoretical considerations. The grid is then extended to ensure that the most promising region of the scan lies away from the boundaries.

Response: The subsection added to Materials and Methods follows:

Parameters for the morphometric approach

For a hard-sphere solvent the physical prefactors for the four terms in the morphometric solvation free energy can be computed using the White Bear functional Mark II [26]. Each term is given as a function depending on the packing fraction η and the solvent radius r_s:

$$\beta p = \frac{\eta}{r_s^3}\frac{3}{4\pi}\left(\frac{1+\eta+\eta^2-\eta^3}{(1-\eta)^3}\right),$$

$$\beta\sigma = \frac{\eta}{r_s^2}\frac{3}{4\pi}\left(-\frac{1+2\eta+8\eta^2-5\eta^3}{3(1-\eta)^3}-\frac{\ln(1-\eta)}{3\eta}\right),$$

$$\beta\kappa = \frac{\eta}{r_s}\frac{3}{4\pi}\left(\frac{4-10\eta+20\eta^2-8\eta^3}{3(1-\eta)^3}+\frac{4\ln(1-\eta)}{3\eta}\right),$$

$$\beta\overline{\kappa} = \eta\frac{3}{4\pi}\left(\frac{-4+11\eta-13\eta^2+4\eta^3}{3(1-\eta)^3}-\frac{4\ln(1-\eta)}{3\eta}\right).$$

Here β is the inverse thermodynamic temperature, which we set to $1.0J^{-1}$, since we are only interested in relative energy levels. Mimicking the properties of water we set $r_s = 1.4$ and $\eta = 0.3665$ (the approximate fluid density relevant to biological processes). This yields the values of $p \approx 0.182\frac{J}{Å^3}$, $\sigma \approx -0.131\frac{J}{Å^2}$, $\kappa \approx 0.112\frac{J}{Å}$ and $\overline{\kappa} \approx -0.03J$.

In Equation 1 we augment the morphometric approach by a linear overlap penalty L to energetically penalize overlapping protein subunits as well as to relax the rigidity of the shapes. The weighting ϕ for L is determined empirically [27].

5. Statistical interpretation of assembly success rates

The reported improvements in assembly success rates (e.g., from 5% to 85%) are striking. However, the statistical interpretation of these values could be improved by including confidence intervals or uncertainty estimates, discussion of run-to-run variability, and additional context regarding the dependence on simulation parameters such as temperature and perturbation size. Since "success rate" is the primary performance metric of the manuscript, a more detailed quantitative discussion would improve the interpretation of the results.

Response: We agree with the reviewer that confidence intervals would improve the interpretation of our results. We therefore add a paragraph at the beginning of the discussion of Fig 4, include the respective intervals in the text, update Tables 2 and 3, and add another paragraph to the discussion of Table 3.

We report the success rate uncertainty as a Wilson score 95% confidence interval [34], which expresses that given the sample size and success rate, we are 95% confident that its true value lies within this interval. We write the interval in square brackets following the success rate.

Table 2:
Comparing success rates. Comparing success rates with confidence intervals of F^*_{sol} and E_{comb} on first states of final phases of successful simulations for both potentials.

	F^*_{sol} touch	E_{comb} touch
F^*_{sol} finish, $T = 3.0$	1%[0.3, 3.6]	6%[3.5, 10.2]
E_{comb} finish, $T = 3.0$	25%[19.5, 31.4]	63%[56.1, 69.4]
E_{comb} finish, $T = 5.0$	36%[29.7, 42.8]	54%[47.1, 60.1]

Table 3:

Protein System	Atoms per subunit	sr [Wilson CI] F^*_{sol}	sr [Wilson CI] $\frac{8}{10}F^*_{sol} + \frac{2}{10}\mathcal{T}_{(1.0,-1.0,1.0)}$	sr [Wilson CI] $\frac{3}{10}F^*_{sol} + \frac{7}{10}\mathcal{T}_{(1.0,-0.4,-0.9)}$
TMV	1206	5.0% [1.4, 16.5]	22.5% [12.3, 37.5]	85.0% [70.9, 92.9]
Hep. B	1134	0.0% [0.0, 8.8]	17.5% [8.7, 32.0]	80.0% [65.2, 89.5]
ORF9b	639	2.5% [0.4, 12.9]	7.5% [2.6, 19.9]	42.5% [28.5, 57.8]
CD40	1113	0.0% [0.0, 8.8]	0.0% [0.0, 8.8]	5.0% [1.4, 16.5]

Response: We further add the following to the discussion of the table:

> *For all systems except CD40, the confidence interval of the best combined potential lies entirely and substantially above that of the pure morphometric approach, indicating that the improvement is not an artifact of the finite number of runs.*

Response: Regarding the dependence on temperature and perturbation size, we note that this is already mapped systematically in the manuscript. Fig 4A reports the success rate as a function of both the simulation temperature T and the rotational perturbation parameter σ_R, and Fig 4C–F report the success-rate landscape over temperature ($T = 3, 4, 5, 6$) and the mixing weight μ. The headline numbers are the best values over these scans, and the heatmaps show that high success rates are obtained over a contiguous region of parameter space rather than at an isolated point. The translational perturbation was held fixed at $\sigma_t = 1.25\,\text{Å}$ throughout.

Minor Comments

1. The description of persistent homology may be difficult for a broad audience. A more intuitive explanation in the main text would improve accessibility.
2. Figure 1 should more clearly explain the connection between the alpha filtration and the resulting topological potential.

Response: We agree with the reviewer that the description of persistent homology is brief in the initial version of the manuscript and that a more intuitive explanation would greatly increase readability and interpretability. We therefore redo Figure 1, which now illustrates the alpha filtration as well as the resulting persistence barcode and how the persistence bar lengths depend on the geometry of the configuration.

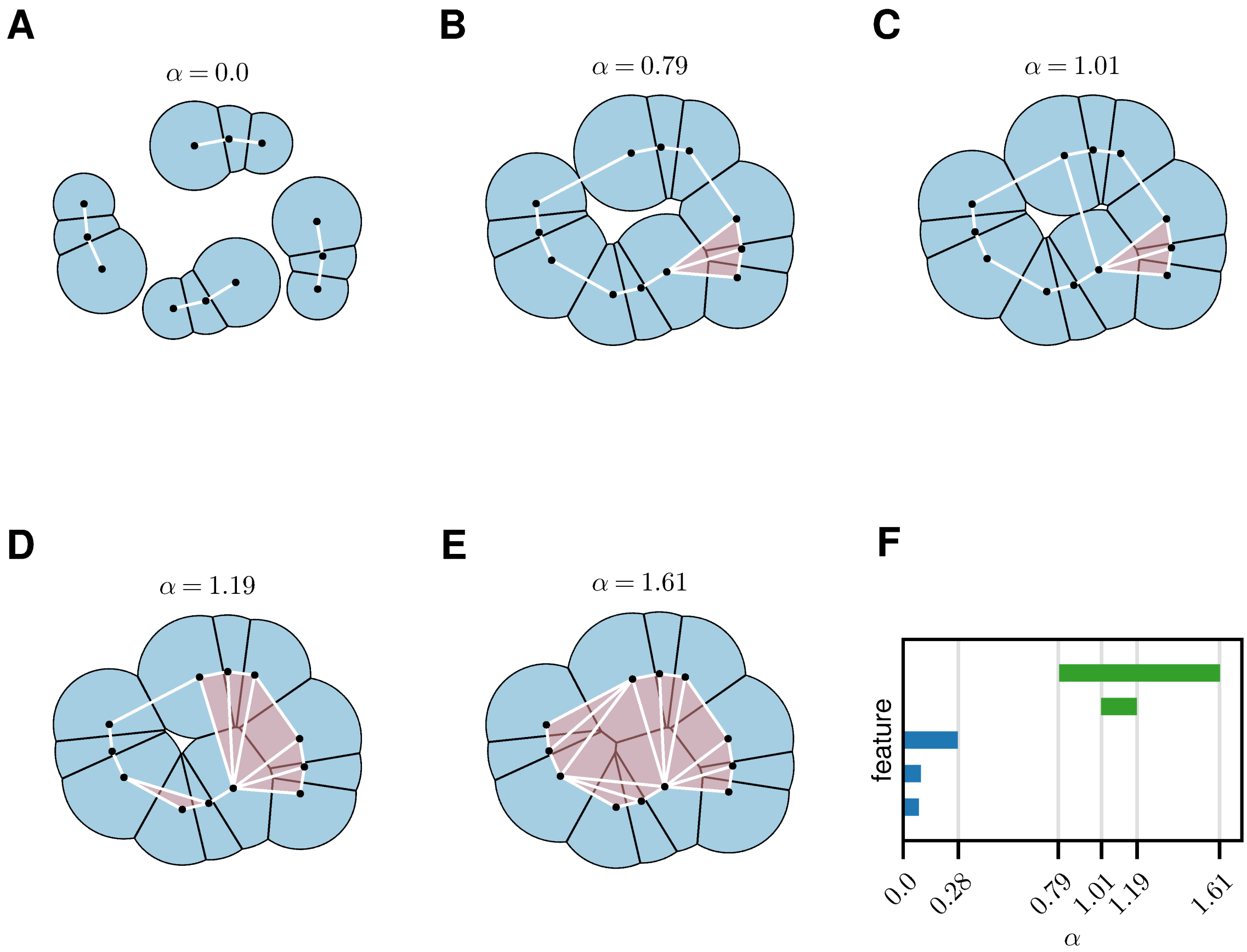

Response: New caption for Figure 1:

> ***Alpha filtration and persistence.*** *Weighted Alpha complexes for a system of four toy shapes in two dimensions at increasing values of α (A-E). Vertices are shown in black, edges in white and triangles in red; the dual intersection of the balls with the weighted Voronoi diagram is shown in blue. At $\alpha = 0$ the shapes carry their solvent-accessible radii (A); as α grows, the space between them is progressively filled (B-E). Panel F shows the resulting persistence barcode: each bar is a topological feature and runs from its birth b to its death d along the α-axis. Blue bars are zero-dimensional features (connected components) and green bars one-dimensional features (loops). The length of a bar, $d - b$, is its persistence, and the total persistence P_i is the sum of the lengths of all i-dimensional bars. We only show the features of finite persistence.*

Response: New paragraph, added after the definition of the topological potential in Eq 2:

> *Consider the persistence barcode in Fig 1F. As the scale α increases, the union of balls grows: initially separate components merge, and loops between the shapes form and later fill in. Each such feature is recorded as a bar from its birth b, the scale at which it appears, to its death d, the scale at which it disappears, and its persistence $d - b$ measures the range of scales over which it survives. Concretely, the long green bar corresponds to the central loop that forms in panel B and persists until it is filled in panel E, while the short green bar corresponds to the short-lived loop that appears to the right of it in panel C and closes again by panel D. The total persistence P_i in Equation 2 is exactly the summed length of all finite persistence i-dimensional bars, so*

$\mathcal{T}$ is a weighted readout of this barcode. The zero-dimensional persistence P_0 is small when the distinct molecules are close together. P_1 and P_2 track the total persistence of loops and voids enclosed between the molecules. Note that no voids exist in the two-dimensional example in Fig 1. Total persistence of loops and voids can be large when few features persist for a long time or many features persist for a short time. The weights λ_i tune how strongly these terms are maximized or minimized during assembly.

3. The discussion of the morphometric solvation model should include a brief intuitive explanation of the integrated mean curvature C and integrated Gaussian curvature X of the solvent-accessible surface.

Response: In the same spirit as the previous remarks, we agree with the reviewer that intuitive explanations of the morphometric solvation model, Random Walk Metropolis, birth-death pairs and the Pearson correlation coefficient would improve readability. We therefore add the following paragraph after the first mention of the morphometric solvation model:

The excluded volume V describes the volume inaccessible to solvent molecule centers. The surface area A captures the area available to solvent molecule centers as close to the solute as possible. The integrated mean curvature C measures the total bending of the solvent-accessible surface. The integrated Gaussian curvature X is a topological measure equal to 4π times the Euler characteristic of the union of balls, hence measuring connectivity, loops and voids of the configuration.

4. The manuscript states that simulations are driven by a Random Walk Metropolis algorithm, but the explanation in the main text is brief. Adding a short description of how moves are proposed and accepted/rejected would improve readability.

Response: ... and a paragraph introducing the Random Walk Metropolis algorithm:

Random Walk Metropolis explores the configuration space by perturbing the current configuration to propose a new one. A proposal that lowers the energy is always accepted, whereas a proposal that raises the energy is accepted only with a probability that decreases as the energy increase grows, and is otherwise rejected. This makes it a powerful algorithm for exploring the configuration space and locating thermodynamically stable states. It does not, however, model dynamics, and no kinetic accessibility between successive states can be inferred from the sequence of configurations it produces.

5. The definition of total persistence should include a simple illustrative example explaining the physical or geometric meaning of the birth (b) and death (d) values within the filtration.

Response: An explanation is included with the answer to minor comments 1. and 2.

6. The manuscript introduces Pearson correlation coefficients between different quantities. A short explanation of what the Pearson coefficient measures, and why it is informative in this context, would improve clarity for a broader audience.

Response: We add the following sentence where the Pearson correlation coefficient is first introduced:

The Pearson coefficient measures the strength and direction of the linear relationship between two quantities, ranging from -1 (perfectly anti-correlated) through 0 (no linear relationship) to $+1$ (perfectly correlated). Here it is informative since it indicates that the topological potential is correlated to d_{RMSD}, pointing toward an exploitable signal to drive simulations toward the assembled state. It furthermore indicates which signs for the weights for total persistence per dimension are likely appropriate.

7. The distinction between thermodynamic accuracy and kinetic accessibility is important and could be emphasized earlier in the manuscript.

Response: We again agree with the reviewer. The paragraph we add in response to minor comment 4 above now introduces this distinction earlier and more prominently.

8. Some notation associated with persistence measures and weighting coefficients could be simplified for readability.

Response: We agree with the reviewer that the notation is slightly verbose, but maintain that the explicitness outweighs the readability cost.

Reviewer #2

The paper introduces a new computational method to improve simulations of protein self-assembly. The key problem is that existing simulations are driven by a short-range geometric energy function known as morphometric approach. It frequently gets stuck in incorrect configurations because the energy landscape is largely flat at long range but rugged up close. To address this, the authors introduce a long-range "topological potential" derived from persistent homology, a mathematical tool that tracks how the shape of the protein system evolves across multiple length scales. This potential is combined with the existing geometric term into a single energy function, where the topological term acts as a coarse funnel drawing proteins into roughly the right arrangement, and the geometric term handles precise docking once they are close.

The work is well-motivated, clearly written, and the results across four protein systems are compelling. Therefore, the work is suitable for publication in PLOS Computational Biology; however, several issues need to be addressed or discussed before it can be accepted, and minor revisions are recommended.

Response: We thank the reviewer for the insightful comments. Some of the points raised by this reviewer were also raised by the other reviewer and we refer to our previous answers where appropriate. At times we extend paragraphs introduced in response to the other reviewer. For a complete picture we refer to the updated manuscript.

1. Missing x-axis label in Figure 2

In Figure 2, the x-axis lacks a label.

Response: Figure updated accordingly.

2. Electrostatic interactions

Electrostatic interactions is a major driver in biomolecular aggregation. How does the topological and geometrical terms include electrostatics? If not, is it possible to incorporate electrostatics to this model?

Response: The goal of the simulation was to model the non-polar solvent-driven part of the self-assembly process, which is modeled using the morphometric approach. The topological potential we introduce is a simulation strategy that acts as a long-range shape matching potential. The exact physical interpretation of this potential is not clear, but from the perspective of shape matching, it behaves in a similar way to long-range physical interactions, including electrostatics. We add the following paragraph to the introduction to clarify the raised points:

> *In physical systems long-range interactions such as electrostatics [16] and long-range hydration forces [28] pull the proteins together and correctly align them. The sum of these effects can be interpreted as a long-range shape matching effect. The biasing potential we introduce in the following does not have a direct physical interpretation but acts as a long-range shape matching potential and in this sense is a similar mechanism to long-range physical interactions.*

3. Attractive van der Waals interactions

The repulsive part of van der Waals interactions can be reflected by the excluded volume terms, but how do the attractive van der Waals interactions are captured? If not, which terms/effects attributed to this model lead to attraction between molecules to form molecular assemblies?

Response: This is a very important point to raise and an important gap to close in the manuscript. The geometric model contains no attractive van der Waals term. The solvent is treated as a hard-sphere fluid. The effective attraction that drives assembly is therefore purely entropic. Since the topological potential attracts molecules at short ranges as well, it can be viewed as compensating for this. To make the entropic origin of the geometric potential explicit, we add the following sentence after stating that the solvent is modeled as a hard-sphere fluid:

> *As such, the effective attraction that drives the assembly of the solutes is entropic in origin.*

4. Utility, reliability, and comparison with other methods

The overarching utility of this method, as well as reliability and accuracy is not clear in the manuscript. Comparison with experimental observables, and or other methods like atomistic and coarse grained MD simulations is important.

Response: We agree with the reviewer on the importance of clarifying the utility of the method as well as its reliability and accuracy. We address the relation to experimental observables and reliability in the responses to the other reviewer's comment 5, this reviewer's comment 5, and in the below paragraphs, added to the discussion.
Regarding the utility of the method, we agree it should be stated more clearly in the manuscript and will answer this point together with questions 6 and 8, which also address the question of comparisons to other methods. We adjust and extend the two final paragraphs in the discussion, which follow after a paragraph added in response to the third comment of the other reviewer:

While all systems studied here are homoassemblies of at most three subunits, the method does not depend conceptually on either restriction, and preliminary work including ligands and proteins shows it extends well.

The calculation of the topological potential $\mathcal{T}$ only depends on the geometry of the atoms and the choice for the parameters λ_i. Its integration into a simulation-governing energy function offers a general computational strategy to overcome rugged energy landscapes in shape assembly simulations. Its performance is demonstrated here across diverse protein systems. The method recovers the thermodynamically accurate states observed in nature with high probability given enough iterations to scan the configuration space. Hence it can be utilized to find low energy states of arbitrary, in particular unknown, molecular assemblies. It retains physical realism in the form of thermodynamic accuracy, but does not model dynamics like molecular dynamics (MD) simulations and therefore does not allow for the inference of kinetic accessibility. However, similarly to MD methods the approach does not depend on prior knowledge of the target states similar to the modeled system. This is in contrast to deep-learning methods like AlphaFold3, whose accuracy on docking tasks has recently been shown to depend strongly on the similarity of the target to the training set [39]. While the mechanism by which the topological potential guides assembly is not yet fully understood, the potential itself is transparent: it is a weighted sum of the total persistence of a weighted alpha complex, a visualizable geometric construction governed by atom geometry and three parameters λ_i. In this sense it is not a black box in the way a deep neural network is, whose behavior is determined by billions of parameters and is correspondingly difficult to interpret. The topological potential by itself can be viewed as a long-range shape-alignment and matching potential, making it useful for simulations in computational biology and materials science.

5. Visual snapshots of experimental structures

In sections where comparisons are made with experimentally determined structures, it would be valuable to include visual snapshots of the experimental structures alongside the model predictions. This would facilitate a more intuitive and direct visual assessment of structural agreement or discrepancy.

Response: We agree with the reviewer's general assessment. For the TMV case: Figure 3 A-D show all experimental structures of TMV subunits relevant to the discussion in the text and are referenced at the beginning of the results section. The results for other systems shown in Figure 6 have a d_{RMSD} of about 1 Å with one of the subunits perfectly matched. They are therefore visually indistinguishable from the experimental state. This should be made apparent, however, and we add the following remark to the text when first referencing the experimental states.

The simulation results are at a distance to the experimental assemblies of $d_{RMSD} \approx 1$ Å. The rendered assemblies are therefore visually indistinguishable from the experimentally determined structures, which is why we do not show the latter separately.

6. Heteroassemblies

Although heteroassemblies are acknowledged as a limitation of the current method, it is not clear why they were not tested, even in a preliminary capacity. Given their biological relevance, the authors should provide a more detailed justification for this omission and, if feasible, consider including at least an exploratory analysis or a discussion of the specific challenges involved.

Response: An important point that we answered in our response to comment 4.

7. Distance heatmap units and color bar

In the distance heatmap, the units of distance are not specified, and the color bar lacks appropriate labels. Please add axis units and a clearly labeled color scale to make the figure fully interpretable without reference to the main text.

Response: We agree with the reviewer's point that the figure should be readable without reference to the main text. The heatmap axes are the weights λ_1 and λ_2, which are dimensionless, and the color encodes $d_z = \log(\mathcal{T}_z(S) - \mathcal{T}_z(S_z))$, the logarithm of a difference in topological potential. It is likewise dimensionless. As such we believe the figure can remain as it is now, but we adjust the caption of the figure so all relevant information is provided together with the figure.

> ***Topological potential of correct assemblies versus $\mathcal{T}$ minimizers.*** *Agreement between correctly assembled reference states and the minimizers of the topological potential $\mathcal{T}$ across the weight parameter space. Render of the two correctly assembled TMV subunits used as the ground truth in the two-subunit case (A). Renders of the three configurations of three TMV subunits that we consider correctly assembled (B-D). Heatmaps over the weight plane (λ_1, λ_2), with $\lambda_0 = 1.0$ fixed (E,F). The color encodes $d_z = \log(\mathcal{T}_z(S) - \mathcal{T}_z(S_z))$, the logarithm of the difference in topological potential between a correct assembly S and the minimizer S_z found at weights $z = (\lambda_0, \lambda_1, \lambda_2)$. Darker colors indicate a smaller difference, i.e. minimizers whose topological potential lies closer to that of the correct assembly. Groups of topological potential minimizers corresponding to the marked regions of the heatmaps (G-N); the minimizers closest to the correct assembly are J and L.*

8. Comparison with structure prediction tools (e.g., AlphaFold3)

A comparison with state-of-the-art structure prediction tools such as AlphaFold3 would considerably strengthen the manuscript and help contextualize the performance of the proposed method. If a direct computational comparison is not feasible within the scope of this work, the authors should at minimum include a substantive discussion of how their approach relates to, complements, or differs from such tools in terms of methodology, applicability, and expected performance.

Response: Another important point to raise, which we answer in the same paragraph as comments 4 and 6.
