# Supplementary material for: Topological potentials guiding protein self-assembly
Source: PLoS Comput Biol. 2026 Sep 3;22(9):e1014709. doi: 10.1371/journal.pcbi.1014709 (PMC13577540; doi:10.1371/journal.pcbi.1014709)
Supplement: Peer review history

# Peer review: Decision Letter 0

**DOI:** 10.1371/journal.pcbi.1014709.r001

**Authors:** Arne Elofsson, Jianhan Chen

21 May 2026

Topological potentials guiding protein self-assembly

Dear Dr. Spirandelli,

Thank you for submitting your manuscript to PLOS Computational Biology. After careful consideration, we feel that it has merit but does not fully meet PLOS Computational Biology's publication criteria as it currently stands. Therefore, we invite you to submit a revised version of the manuscript that addresses the points raised during the review process.

Please submit your revised manuscript by Jul 21 2026 11:59PM. If you will need more time than this to complete your revisions, please reply to this message or contact the journal office at ploscompbiol@plos.org. When you're ready to submit your revision, log on to https://www.editorialmanager.com/pcompbiol/ and select the 'Submissions Needing Revision' folder to locate your manuscript file.

Please include the following items when submitting your revised manuscript:

* A letter that responds to each point raised by the editor and reviewer(s). You should upload this letter as a separate file labeled 'Response to Reviewers'. This file does not need to include responses to formatting updates and technical items listed in the 'Journal Requirements' section below.

* A marked-up copy of your manuscript that highlights changes made to the original version. You should upload this as a separate file labeled 'Revised Manuscript with Track Changes'.

* An unmarked version of your revised paper without tracked changes. You should upload this as a separate file labeled 'Manuscript'.

We look forward to receiving your revised manuscript.

Kind regards,

Jianhan Chen, Ph.D.

Academic Editor

PLOS Computational Biology

Arne Elofsson

Section Editor

PLOS Computational Biology

**Additional Editor Comments:**

Apologies for the delay. We were waiting for a third reviewer who did not submit the report after the due date despite several reminders. Both reviewers clearly appreciate the innovation and importance of this work, while raising various minor and major concerns on a range of aspects. I will greatly appreciate a revised manuscript with all points thoroughly addressed. Thank you.

**Journal Requirements:**

1) Please ensure that the CRediT author contributions listed for every co-author are completed accurately and in full.

At this stage, the following Authors/Authors require contributions: Ivan Lothar Arbo Spirandelli. Please ensure that the full contributions of each author are acknowledged in the "Add/Edit/Remove Authors" section of our submission form.

The list of CRediT author contributions may be found here: https://journals.plos.org/ploscompbiol/s/authorship#loc-author-contributions

2) We ask that a manuscript source file is provided at Revision. Please upload your manuscript file as a .doc, .docx, .rtf or .tex. If you are providing a .tex file, please upload it under the item type u2018LaTeX Source Fileu2019 and leave your .pdf version as the item type u2018Manuscriptu2019.

3) Please upload all main figures as separate Figure files in .tif or .eps format. For more information about how to convert and format your figure files please see our guidelines:

https://journals.plos.org/ploscompbiol/s/figures

4) We have noticed that you have uploaded Supporting Information files, but you have not included a list of legends. Please add a full list of legends for your Supporting Information files after the references list.

5) Please amend your detailed Financial Disclosure statement. This is published with the article. It must therefore be completed in full sentences and contain the exact wording you wish to be published.

- State the initials, alongside each funding source, of each author to receive each grant. For example: "This work was supported by the National Institutes of Health (####### to AM; ###### to CJ) and the National Science Foundation (###### to AM)."

- State what role the funders took in the study. If the funders had no role in your study, please state: "The funders had no role in study design, data collection and analysis, decision to publish, or preparation of the manuscript.".

If you did not receive any funding for this study, please simply state: u201cThe authors received no specific funding for this work.u201d

**Reviewers' comments:**

Reviewer's Responses to Questions

**Comments to the Authors:**

**Please note here if the review is uploaded as an attachment.**

Reviewer #1: The authors study protein self-assembly using a computational approach that combines a morphometric geometric solvation model with a long-range topological potential derived from persistent homology. The central idea is to use weighted total persistence as an energetic bias to guide assembly pathways and reduce kinetic trapping in rugged energy landscapes. The authors apply the approach to several systems, including TMV dimers, hepatitis B core protein, SARS-CoV-2 ORF9b, and CD40 ligand, and report substantial improvements in assembly success rates compared to the geometric model alone.

The manuscript presents an interesting and potentially useful approach for improving simplified assembly simulations. The reported increases in assembly success rates are substantial, and the paper is generally clearly written. However, several points need to be addressed before publication.

1. The physical interpretation of the topological potential remains unclear. While the authors briefly relate a specific parameter choice to Gaussian curvature, the manuscript does not provide clear physical intuition regarding whether the topological term represents an effective approximation of omitted long-range interactions (e.g., electrostatic or hydrophobic effects) or functions primarily as an algorithmic bias that reshapes the energy landscape to reduce kinetic trapping. Relatedly, the manuscript frames protein assembly simulations as being dominated by short-range nonpolar interactions, whereas many assembly processes are strongly influenced by electrostatics, hydrogen bonding, and other longer-range effects. Since electrostatic interactions are omitted entirely, it remains unclear how the proposed topological potential compares to more conventional implicit-solvent or coarse-grained models that include electrostatics, and whether the topological term provides fundamentally new information or instead compensates for omitted physical interactions.

2. The manuscript focuses on relatively small homo-oligomeric systems, primarily dimers and trimers, rather than full viral capsids or larger supramolecular assemblies. Thus, the reported increases in “assembly success rates” correspond to improved formation of small native-like oligomeric complexes rather than full virus assembly. It would be useful for the authors to discuss more explicitly whether the proposed topological potential is expected to extend to larger-scale coarse-grained assembly simulations involving tens to hundreds of subunits, such as HBV or TMV capsid assembly. In particular, it remains unclear whether persistent homology can be computed efficiently using coarse-grained capsomers or bead-based representations rather than atomistic coordinates, whether the topological potential is expected to guide global shell closure or suppress malformed intermediates during capsid assembly, and how the computational cost is expected to scale with increasing system size.

3. Relatedly, the present model treats protein subunits as rigid bodies. In contrast, conformational flexibility often plays an important role in viral assembly pathways and polymorphism, including in coarse-grained models capable of assembling complete viral shells. See, for example, “From Disorder to Icosahedral Symmetry: How Conformation-Switching Subunits Enable RNA Virus Assembly,” Science Advances 11, eady7241 (2025). The authors should discuss whether the proposed topological framework could eventually be combined with flexible coarse-grained models and whether it is intended as a complementary guiding potential for improving existing coarse-grained assembly simulations rather than as a standalone assembly model.

4. The topological potential depends on weighting coefficients (λ0,λ1,λ2), which appear to be selected empirically through parameter scans, but the procedure used to choose these parameters remains somewhat unclear. The authors should clarify how the λi\lambda_iλi values are selected, whether they are optimized systematically or motivated by physical considerations, and how sensitive the results are to these choices. The apparent transferability across systems is interesting, but a more systematic robustness analysis would improve the conclusions.

Similarly, the implementation of Eq. (1) remains somewhat vague. The manuscript should more clearly state the numerical values used for the morphometric parameters, how these parameters are selected, and whether any of them are adjusted during the simulations. This clarification is particularly important for readers unfamiliar with morphometric approaches.

5. The reported improvements in assembly success rates (e.g., from 5% to 85%) are striking. However, the statistical interpretation of these values could be improved by including confidence intervals or uncertainty estimates, discussion of run-to-run variability, and additional context regarding the dependence on simulation parameters such as temperature and perturbation size. Since “success rate” is the primary performance metric of the manuscript, a more detailed quantitative discussion would improve the interpretation of the results.

Minor Comments

1. The description of persistent homology may be difficult for a broad audience. A more intuitive explanation in the main text would improve accessibility.

2. Figure 1 should more clearly explain the connection between the alpha filtration and the resulting topological potential.

3. The discussion of the morphometric solvation model should include a brief intuitive explanation of the integrated mean curvature C and integrated Gaussian curvature X of the solvent-accessible surface.

4. The manuscript states that simulations are driven by a Random Walk Metropolis algorithm, but the explanation in the main text is brief. Adding a short description of how moves are proposed and accepted/rejected would improve readability.

5. The definition of total persistence should include a simple illustrative example explaining the physical or geometric meaning of the birth (b) and death (d) values within the filtration.

6. The manuscript introduces Pearson correlation coefficients between different quantities. A short explanation of what the Pearson coefficient measures, and why it is informative in this context, would improve clarity for a broader audience.

7. The distinction between thermodynamic accuracy and kinetic accessibility is important and could be emphasized earlier in the manuscript.

8. Some notation associated with persistence measures and weighting coefficients could be simplified for readability.

Reviewer #2: the review is uploaded as an attachment

**********

**Have the authors made all data and (if applicable) computational code underlying the findings in their manuscript fully available?**

The PLOS Data policy requires authors to make all data and code underlying the findings described in their manuscript fully available without restriction, with rare exception (please refer to the Data Availability Statement in the manuscript PDF file). The data and code should be provided as part of the manuscript or its supporting information, or deposited to a public repository. For example, in addition to summary statistics, the data points behind means, medians and variance measures should be available. If there are restrictions on publicly sharing data or code —e.g. participant privacy or use of data from a third party—those must be specified.

Reviewer #1: Yes

Reviewer #2: Yes

**********

PLOS authors have the option to publish the peer review history of their article (what does this mean?). If published, this will include your full peer review and any attached files.

If you choose “no”, your identity will remain anonymous but your review may still be made public.

**Do you want your identity to be public for this peer review?**  For information about this choice, including consent withdrawal, please see our Privacy Policy.

Reviewer #1: No

Reviewer #2: **Yes:** Vysakh Ramachandran

[NOTE: If reviewer comments were submitted as an attachment file, they will be attached to this email and accessible via the submission site. Please log into your account, locate the manuscript record, and check for the action link "View Attachments". If this link does not appear, there are no attachment files.]

**Figure resubmission:**

While revising your submission, we strongly recommend that you use PLOS’s NAAS tool (https://ngplosjournals.pagemajik.ai/artanalysis) to test your figure files. NAAS can convert your figure files to the TIFF file type and meet basic requirements (such as print size, resolution), or provide you with a report on issues that do not meet our requirements and that NAAS cannot fix.

After uploading your figures to PLOS’s NAAS tool - https://ngplosjournals.pagemajik.ai/artanalysis, NAAS will process the files provided and display the results in the "Uploaded Files" section of the page as the processing is complete. If the uploaded figures meet our requirements (or NAAS is able to fix the files to meet our requirements), the figure will be marked as "fixed" above. If NAAS is unable to fix the files, a red "failed" label will appear above. When NAAS has confirmed that the figure files meet our requirements, please download the file via the download option, and include these NAAS processed figure files when submitting your revised manuscript.

**Reproducibility:**

?>

Attachment Submitted filename: Review_Questions.pdf

# Author comment: Author response to Decision Letter 1

**DOI:** 10.1371/journal.pcbi.1014709.r002

22 Jul 2026

Attachment Submitted filename: response_to_reviewers.pdf

# Peer review: Decision Letter 1

**DOI:** 10.1371/journal.pcbi.1014709.r003

**Authors:** Nir Ben-Tal, Jianhan Chen

11 Aug 2026

Dear Dr. Spirandelli,

We are pleased to inform you that your manuscript 'Topological potentials guiding protein self-assembly' has been provisionally accepted for publication in PLOS Computational Biology.

Before your manuscript can be formally accepted you will need to complete some formatting changes, which you will receive in a follow up email. A member of our team will be in touch with a set of requests.

Please note that your manuscript will not be scheduled for publication until you have made the required changes, so a swift response is appreciated.

IMPORTANT: The editorial review process is now complete. PLOS will only permit corrections to spelling, formatting or significant scientific errors from this point onwards. Requests for major changes, or any which affect the scientific understanding of your work, will cause delays to the publication date of your manuscript.

Should you, your institution's press office or the journal office choose to press release your paper, you will automatically be opted out of early publication. We ask that you notify us now if you or your institution is planning to press release the article. All press must be co-ordinated with PLOS.

Thank you again for supporting Open Access publishing; we are looking forward to publishing your work in PLOS Computational Biology.

Best regards,

Jianhan Chen, Ph.D.

Academic Editor

PLOS Computational Biology

Nir Ben-Tal

Section Editor

PLOS Computational Biology

***********************************************************

Reviewer's Responses to Questions

**Comments to the Authors:**

**Please note here if the review is uploaded as an attachment.**

Reviewer #1: The authors have addressed all my concerns and comments, and those of the other referee. Thus, I recommend the paper for publication.

Reviewer #2: All of my concerns have been addressed in the revised manuscript. The authors have responded appropriately to the reviewers' comments, and I believe the manuscript has improved significantly. I have no further concerns and recommend that the manuscript be accepted for publication.

**********

**Have the authors made all data and (if applicable) computational code underlying the findings in their manuscript fully available?**

The PLOS Data policy requires authors to make all data and code underlying the findings described in their manuscript fully available without restriction, with rare exception (please refer to the Data Availability Statement in the manuscript PDF file). The data and code should be provided as part of the manuscript or its supporting information, or deposited to a public repository. For example, in addition to summary statistics, the data points behind means, medians and variance measures should be available. If there are restrictions on publicly sharing data or code —e.g. participant privacy or use of data from a third party—those must be specified.

Reviewer #1: Yes

Reviewer #2: Yes

**********

PLOS authors have the option to publish the peer review history of their article (what does this mean?). If published, this will include your full peer review and any attached files.

If you choose “no”, your identity will remain anonymous but your review may still be made public.

**Do you want your identity to be public for this peer review?**  For information about this choice, including consent withdrawal, please see our Privacy Policy.

Reviewer #1: No

Reviewer #2: **Yes:** Vysakh Ramachandran

# Editor report: Acceptance letter

**DOI:** 10.1371/journal.pcbi.1014709.r004

**Authors:** Nir Ben-Tal, Jianhan Chen

PCOMPBIOL-D-26-00899R1

Topological potentials guiding protein self-assembly

Dear Dr Spirandelli,

I am pleased to inform you that your manuscript has been formally accepted for publication in PLOS Computational Biology. Your manuscript is now with our production department and you will be notified of the publication date in due course.

The corresponding author will soon be receiving a typeset proof for review, to ensure errors have not been introduced during production. Please review the PDF proof of your manuscript carefully, as this is the last chance to correct any errors. Please note that major changes, or those which affect the scientific understanding of the work, will likely cause delays to the publication date of your manuscript.

Soon after your final files are uploaded, unless you have opted out, the early version of your manuscript will be published online. The date of the early version will be your article's publication date. The final article will be published to the same URL, and all versions of the paper will be accessible to readers.

For Research, Software, and Methods articles, you will receive an invoice from PLOS for your publication fee after your manuscript has reached the completed accept phase. If you receive an email requesting payment before acceptance or for any other service, this may be a phishing scheme. Learn how to identify phishing emails and protect your accounts at https://explore.plos.org/phishing.

Thank you again for supporting PLOS Computational Biology and open-access publishing. We are looking forward to publishing your work!

With kind regards,

Sharmila Kamatchi

PLOS Computational Biology | Carlyle House, Carlyle Road, Cambridge CB4 3DN | United Kingdom ploscompbiol@plos.org | Phone +44 (0) 1223-442824 | ploscompbiol.org | @PLOSCompBiol
